# Identification of 7-Ketocholesterol-Modulated Pathways and Sterculic Acid Protective Effect in Retinal Pigmented Epithelium Cells by Using Genome-Wide Transcriptomic Analysis

**DOI:** 10.3390/ijms24087459

**Published:** 2023-04-18

**Authors:** Ana Pariente, Álvaro Pérez-Sala, Rodrigo Ochoa, Miriam Bobadilla, Ángela Villanueva-Martínez, Rafael Peláez, Ignacio M. Larráyoz

**Affiliations:** 1 Biomarkers and Molecular Signaling Group, Neurodegeneration Area, Center for Biomedical Research of La Rioja (CIBIR), Piqueras 98, 26006 Logroño, Spain; apariente@riojasalud.es (A.P.); aperez@riojasalud.es (Á.P.-S.); rochoa.iacs@aragon.es (R.O.); mbobadilla@riojasalud.es (M.B.); avmartinez@riojasalud.es (Á.V.-M.); 2 Proteomics Research Core Facility, Aragonese Institute of Health Sciences (IACS), University of Zaragoza, San Juan Bosco 13, 50009 Zaragoza, Spain; 3 Biomarkers, Artificial Intelligence and Signaling (BIAS), Department of Nursing, University of La Rioja, Duquesa de la Victoria 88, 26006 Logroño, Spain

**Keywords:** 7-ketocholesterol, sterculic acid, AMD, transcriptome, retina

## Abstract

Age-related macular degeneration (AMD) is the leading cause of blindness in developed countries. AMD is characterized by the formation of lipidic deposits between the retinal pigment epithelium (RPE) and the choroid called drusen. 7-Ketocholesterol (7KCh), an oxidized-cholesterol derivative, is closely related to AMD as it is one of the main molecules accumulated in drusen. 7KCh induces inflammatory and cytotoxic responses in different cell types, and a better knowledge of the signaling pathways involved in its response would provide a new perspective on the molecular mechanisms that lead to the development of AMD. Furthermore, currently used therapies for AMD are not efficient enough. Sterculic acid (SA) attenuates the 7KCh response in RPE cells and is presented as an alternative to improve these therapies. By using genome-wide transcriptomic analysis in monkey RPE cells, we have provided new insight into 7KCh-induced signaling in RPE cells, as well as the protective capacity of SA. 7KCh modulates the expression of several genes associated with lipid metabolism, endoplasmic reticulum stress, inflammation and cell death and induces a complex response in RPE cells. The addition of SA successfully attenuates the deleterious effect of 7KCh and highlights its potential for the treatment of AMD.

## 1. Introduction

Age-related macular degeneration (AMD) is a serious disease affecting the macula of the retina, and it is characterized by a gradual loss of central vision because of aging. AMD represents, in developed countries, the principal cause of blindness in people older than 65 years [1,2,3,4]. This disease is mainly classified into two different types: dry AMD and wet AMD [1,5,6]. Dry AMD is the most common form of the disease and is characterized by the accumulation of yellowish extracellular deposits called drusen [6,7,8,9]. Because of aging, intracellular material not digested by the retinal pigment epithelium (RPE) begins to accumulate around it, forming these specific deposits [7,8,10]. Drusen can be formed both in the apical and basolateral part of the RPE, but only drusen located basolaterally, between the epithelium and the choroid, are characteristic of AMD and represent the first hallmark of the disease [7,8,9]. For this reason, an important part of the research related to AMD is centered on the RPE, as it is in this area of the retina where the disease can be detected earlier. Nevertheless, some studies have been focused on other parts of the retina, such as the photoreceptors, the choroid or the extracellular cell matrix of the RPE [11,12,13]. When the number and size of drusen increases, the molecules inside them—mainly lipid compounds, cholesterol derivatives and unfolded-oxidized proteins—trigger different responses that may damage the epithelium, promoting the development of dry AMD [3,8,10,14,15]. Dry AMD does not usually cause complete loss of central vision due to its slow progress [3].

On the other hand, wet AMD is the most severe form of the disease. It is characterized by an abnormal angiogenesis process of choroidal blood vessels from the macula called choroidal neovascularization (CNV) [1,3,6,11]. Eventually, this process leads to blood and fluid leakage that can scar the macula, leading to a rapid and permanent loss of central vision [5,6]. Nowadays, anti-VEGF therapy is the only treatment available for wet AMD patients to counteract CNV. However, these therapies are not efficient enough, as they are not exempt from serious side effects, and some patients develop resistance to these drugs [1,5,6,16,17,18]. For this reason, there is a need to find new alternatives to these therapies by looking for new therapeutic targets.

7-Ketocholesterol (7KCh) is an oxidized cholesterol derivative or oxysterol known for inducing oxidative stress, inflammation and cell death in different cell types, including retinal cells [15,19,20,21,22,23,24,25,26,27,28]. There is strong evidence suggesting a connection between 7KCh and AMD since this oxysterol is formed and accumulated in drusen as a consequence of aging [29,30,31,32,33,34]. In addition, 7KCh has been related to other neurodegenerative diseases that, like AMD, are characterized by the formation of specific deposits, such as atherosclerosis or Alzheimer’s disease [4,29]. The response induced by 7KCh seems to depend on the cell type and dose used, and little is known about its mechanism of action in retinal cells [35]. The study of the signaling pathways involved in the response to 7KCh in RPE cells could provide a new perspective on the molecular mechanisms that lead to the development of AMD. 

Likewise, it would be interesting to find a molecule capable of counteracting the deleterious effect of 7KCh in the retina. Sterculic acid (SA) is a natural cyclopropane fatty acid mainly obtained from the seeds of *Sterculia foetida*. SA is known to inhibit the enzyme from de novo lipogenesis SCD1 (Stearoyl coenzyme-A desaturase 1) [36,37,38]. One of the most promising breakthroughs in the last decade regarding SA has been the discovery of its potential as a functional antagonist of 7KCh in the human RPE cell line ARPE-19 [19,20,35]. At low concentrations, SA protected these cells from 7KCh-induced toxicity and inflammation. Moreover, SA was able to reduce in vivo laser-induced CNV levels in a rat model, suggesting that SA could be a good candidate for the treatment of AMD [19,32]. We have previously reported the potential of SA for the treatment of retinal diseases, as it has no toxic effects on RPE cells and regulates crucial pathways related to survival, inflammation and cell death. However, the mechanism by which SA attenuates the 7KCh effect is still poorly understood and seems to be independent of its ability to inhibit SCD1 [39]. Therefore, the study of the mechanism of action of SA in the inhibition of the 7KCh response in RPE cells would be of interest in the improvement of the therapies currently used in AMD.

In this work, genome-wide transcriptomic analysis has been used to unravel the molecular pathways induced by 7KCh as well as the inhibition that SA exerts on the response of this oxysterol in RPE cells. Results showed a wide range of genes altered in response to 7KCh and reaffirmed the potential of SA as a functional antagonist of this oxysterol in the retina. 

## 2. Results

### 2.1. 7KCh Induces Cell Death and Inflammatory Responses in mRPE Cells, Which Are Attenuated by SA Administration

**Figure 1:**
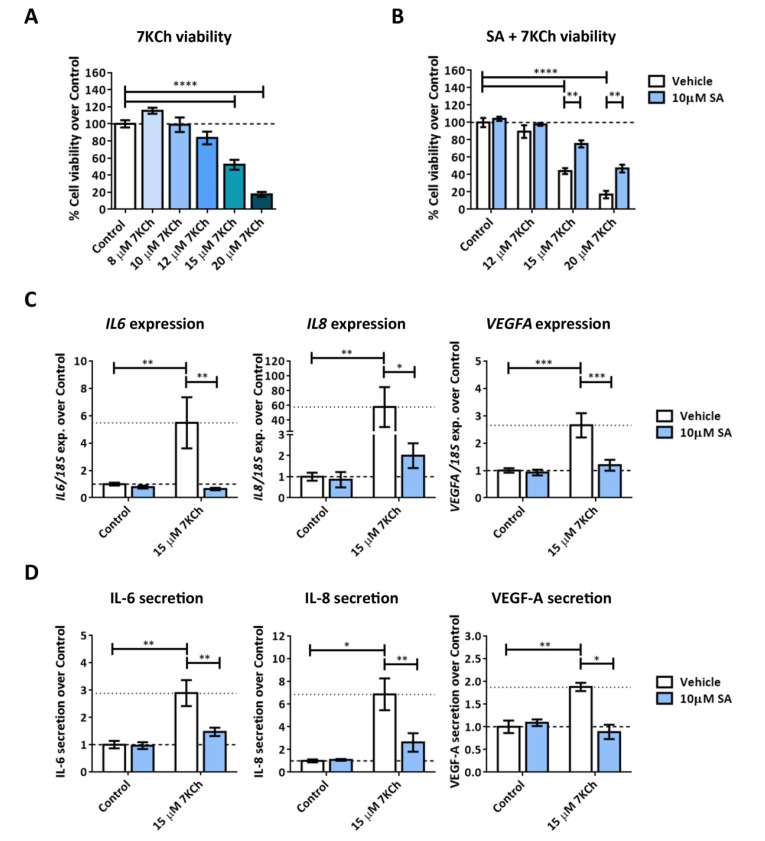
Cell toxicity and inflammation induced by 7KCh and protective effect exerted by SA on mRPE cells. (**A**) Cell viability determined by MTS assay in mRPE cells treated with increasing concentrations of 7KCh or with (**B**) 10 μM SA and 12–20 μM 7KCh for 24 h. (**C**) Quantification by qRT-PCR of *IL6*, *IL8* and *VEGFA* gene expression levels in mRPE cells treated with 15 μM 7KCh and 10 μM SA for 24 h, normalized with respect to rRNA *18S* expression. (**D**) Secreted levels of IL-6, IL-8 and VEGF-A in mRPE cells exposed to 15 μM 7KCh and 10 μM SA for 48 h and measured by ELISA. The vehicle group in the graphs represents the control (control-vehicle) and the 7KCh (7KCh-vehicle) treatment. Data are represented as mean ± SEM of at least three different experiments. The dashed and dotted lines are a guidance mark of control and 7KCh value, respectively. ANOVA test was used for statistical analysis followed by Tukey (in **A**) or Sidak (in **B**–**D**) post hoc test. * *p* < 0.05; ** *p* < 0.01; *** *p* < 0.001; **** *p* < 0.0001.

We treated mRPE (monkey retinal pigment epithelium) cells for 24 h with increasing concentrations of 7KCh (8–20 μM) in order to test its effect on cell viability by MTS assay. At 15 μM, 7KCh produced a decrease of approximately 50% in cell viability, and at 20 μM, the viability was reduced to almost 20% (Figure 1A). 

Co-treatment with 10 μM SA showed a protective effect over 7KCh-induced toxicity even at the highest concentration of 7KCh used (Figure 1B). On the other hand, incubation with 15 μM 7KCh induced inflammation in mRPE cells, which was observed by the increase in the gene expression levels of *IL6*, *IL8* and *VEGFA* after 24 h of treatment (Figure 1C) and by the increase in the secretion of these cytokines to the medium after 48 h (Figure 1D). The addition of 10 μM SA attenuated both the increase in gene expression and secretion of the three cytokines evaluated (Figure 1C,D).

### 2.2. Exposure of mRPE Cells to 7KCh Causes Modulation of Genes Associated with Lipid Metabolism, ER (Endoplasmic Reticulum) Stress, Inflammation and Cell Death

**Figure 2:**
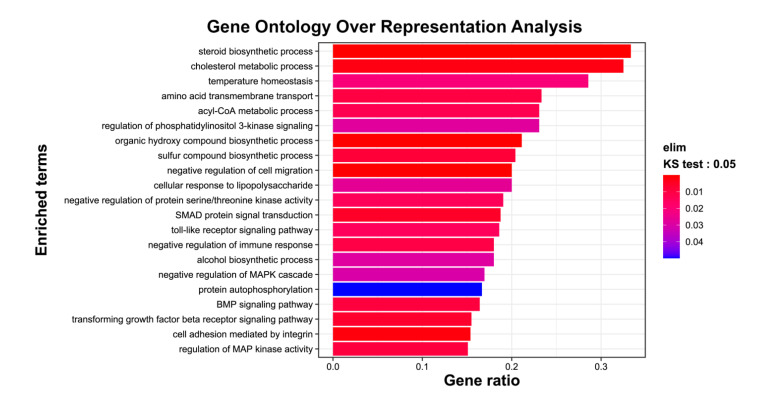
TOP GO categories for biological processes in gene expression altered by 15 μM 7KCh in mRPE cells. DEGs between control and 7KCh-treated cells for 24 h were used to perform Gene Ontology (GO) enrichment analysis. The annotated DEGs were classified into different categories according to GO terms by EnrichGO. Functional groups are shown on the *y*-axis, while the gene ratio of each category is displayed on the *x*-axis.

In order to study the signaling pathways involved in the response of retinal cells to 7KCh, the genetic profile of mRPE cells exposed to this oxysterol was analyzed. Genome-wide transcriptome analysis was performed in mRPE cells treated with 15 μM 7KCh for 24 h to locate differentially expressed genes (DEGs) with respect to control cells. After applying FDR (false discovery rate) methods [40], 757 DEGs were found between 7KCh-treated and control cells (Table S1). TOP GO (Gene Ontology) enrichment analysis showed a wide range of biological processes were modified in response to 15 μM 7KCh exposition (Figure 2). A summary of relevant genes altered by 7KCh in mRPE cells and the associated pathways can be found in Table 1.

First, we found a strong negative regulation over the expression of several genes of fatty acid biosynthesis (*ACACA*, *ACLY*, *CAV1*, *FADS1*, *FADS2*, *FASN*, *SCD*) and, especially, sterol biosynthesis (*FDFT1*, *HMGCR*, *HMGCS1*, *INSIG1*, *LDLR*, *LSS*, *MSMO1*, *MVD*, *MVK*, *SQLE*). Likewise, an increase in the expression levels of genes associated with ER (endoplasmic reticulum) stress was observed (*ASNS*, *ATF3*, *CEBPB*, *CEBPG*, *EIF2S2*, *ERN1*, *GFPT1*, *HSPA5*, *HYOU1*, *PREB*, *SRPRB*, *SSR1*), as well as in the expression of several genes belonging to MAPK and NFκB inflammatory pathways (*CXCL2*, *EGFR*, *FOSB*, *IL1A*, *IL6*, *MAP2K1*, *MAP3K8*, *MYC*, *NFKBIA*, *NFKBIB*, *NFBIZ*, *TRAF1*, *VEGFA*). Lastly, some genes of different cell death pathways were also upregulated by 7KCh treatment (*CASP1*, *DFNA5*, *GADD45A*, *PMAIP1*, *TNFRSF10B*, *TP53I3*). 

### 2.3. SA Exerts a Reverse Effect on the Modulation of Part of the Genes Altered by 7KCh

**Figure 3:**
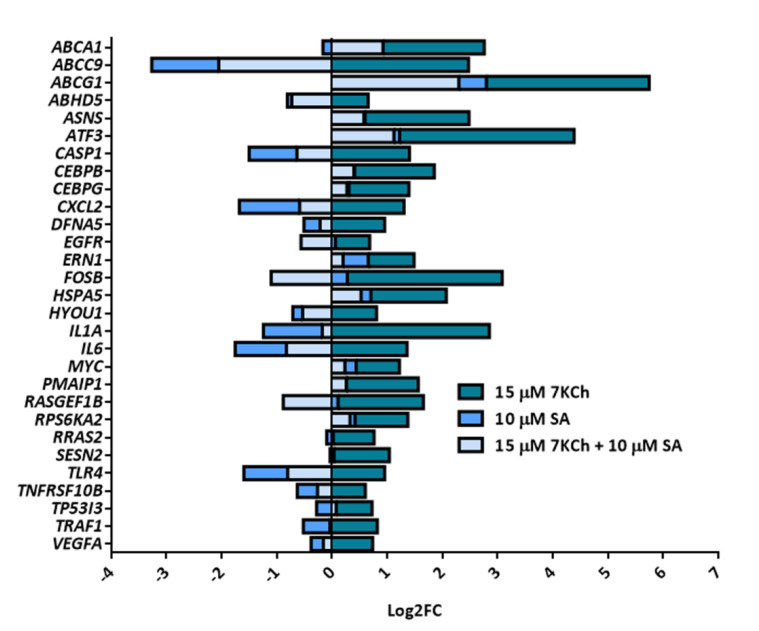
Reversion effect induced by 10 μM SA over several representative genes altered by 15 μM 7KCh. *Y*-axis shows examples of genes modulated by 7KCh, whereas the *X*-axis represents the change in the expression of these genes in mRPE cells treated with 15 μM 7KCh, 10 μM SA or with the combination of SA and 7KCh with respect to the control and expressed as Log2FC. Positive Log2FC represents upregulated genes, while negative Log2FC represents downregulated genes. SA gene modulation data were obtained from our previous work [39].

Genome-wide transcriptome analysis was also performed in mRPE cells treated with the combination of 10 μM SA and 15 μM 7KCh. We obtained 1291 DEGs between the combined treatment and 7KCh alone. Compared with DEGs found between 7KCh-treated and control cells, SA was able to reverse the modulation of 212 genes altered by 7KCh and associated with ER stress, inflammation and cell death (Table S2). Figure 3 summarizes this reversion effect. 

### 2.4. TLR4 Does Not Have an Important Role in Mediating 7KCh-Response in mRPE Cells

**Figure 4:**
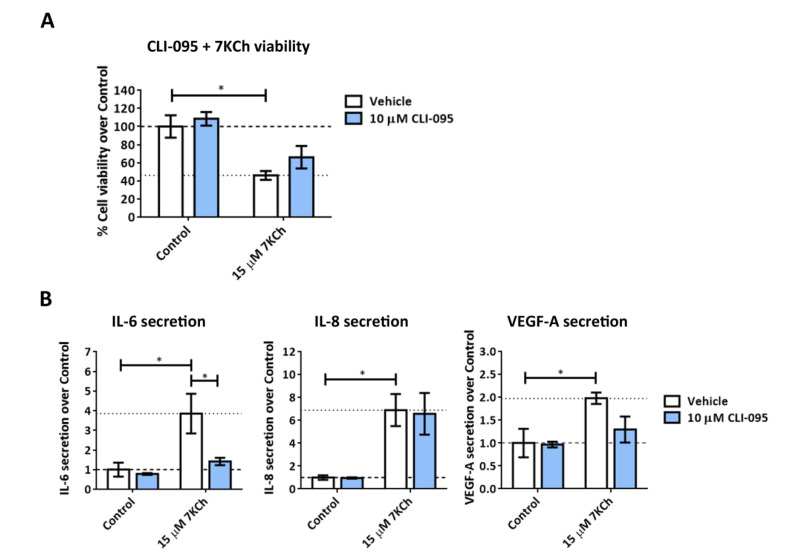
Effect of TLR4 inhibition with CLI-095 on 7KCh-induced toxicity and inflammation. (**A**) Cell viability determined by MTS assay in mRPE cells treated with 15 μM 7KCh and 10 μM CLI-095 for 24 h. (**B**) Secreted levels of IL-6, IL-8 and VEGF-A in mRPE cells exposed to 15 μM 7KCh and 10 μM CLI-095 for 48 h and measured with ELISA. CLI-095 was added with a pretreatment of 2 h with respect to 7KCh. The vehicle group in the graphs represents the control (control-vehicle) and the 7KCh (7KCh-vehicle) treatment. Data are represented as mean ± SEM of at least three different experiments. The dashed and dotted lines are a guidance mark of control and 7KCh value, respectively. ANOVA test was used for statistical analysis followed by Sidak post hoc test. * *p* < 0.05.

TLR4 has been previously proposed as the major receptor mediating 7KCh-response in ARPE-19 cells [20]. In addition, data obtained from transcriptome sequencing in mRPE cells showed an increase in *TLR4* expression. For that reason, we tested the implication of the TLR4 receptor in 7KCh-induced inflammation and cell death in mRPE cells. We treated mRPE cells with 15 μM 7KCh and the specific TLR4 inhibitor CLI-095 at the concentration of 10 μM. No effect was observed over 7KCh-induced toxicity as assessed by MTS assay (Figure 4A). Regarding inflammation, ELISAs showed that TLR4 inhibition was only capable of attenuating IL-6 secretion to the medium.

### 2.5. 7KCh Does Not Induce ROS Release in mRPE Cells

**Figure 5:**
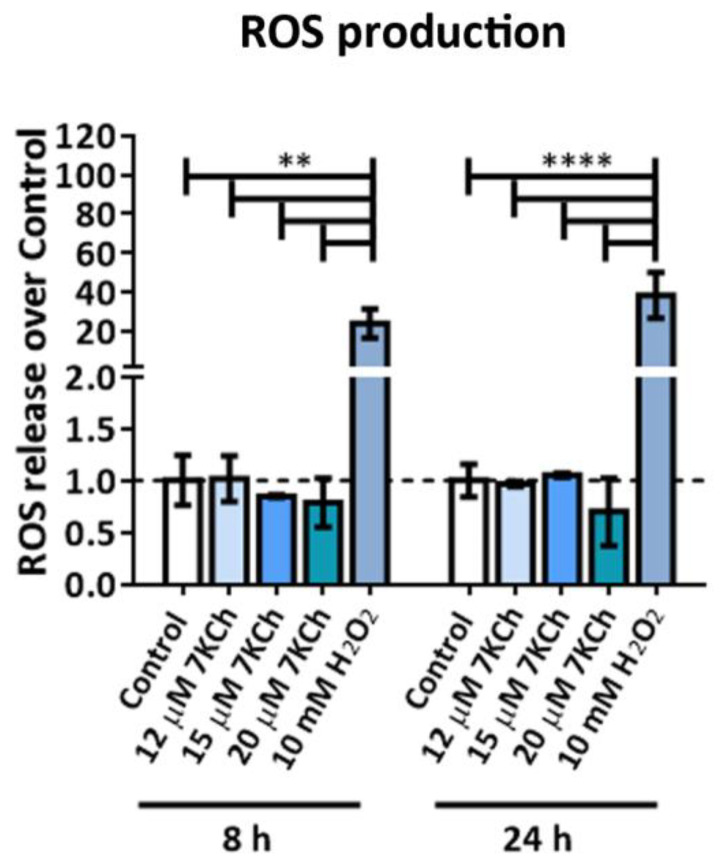
ROS released in mRPE cells exposed to 12–20 μM 7KCh for 8 h and 24 h. ROS production levels were measured by oxidation of H_2_DCFDA probe to DCF, and 10 mM H_2_O_2_ was used as a positive control. Data are represented as mean ± SEM of at least three different experiments. The dashed line is a guidance of the control value. ANOVA test was used for statistical analysis followed by Tukey post hoc test. ** *p* < 0.01; **** *p* < 0.0001.

In order to study the induction of oxidative stress in response to 7KCh, we measured ROS levels in mRPE cells incubated with 12–20 μM 7KCh for 8 h and 24 h. Treatment with 10 mM H_2_O_2_ was used as a positive control [41]. No difference was observed between cells exposed to the oxysterol and non-treated control cells (Figure 5), indicating that the response exerted by 7KCh in retinal cells is independent of ROS production. 

### 2.6. ER Stress Induced by 7KCh in mRPE Cells Is Mediated by the Unfolded Protein Response

Data obtained from the transcriptome analysis showed an upregulation of several genes related to ER stress and, specifically, to the unfolded protein response (UPR). Among these genes, we found *HSPA5*, coding for GRP78, and *ERN1*, coding for IRE1, two important regulators of this response. We also found some genes related to PERK activation, such as *ASNS*, *ATF3* and *CEBPG*, and genes coding for proteins that are accumulated in the ER in response to stress, such as *HYOU1*. Treatment with SA reduced the expression of several of these genes, including *HSPA5*, *HYOU1*, *ERN1*, *CEBPG* and *ATF3* (Figure 3, Table S2).

**Figure 6:**
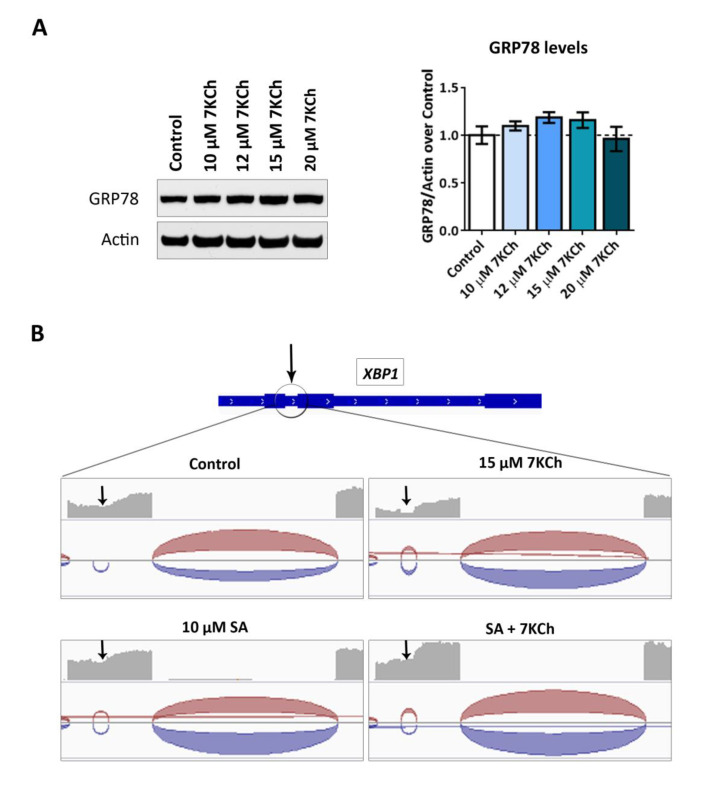
UPR signaling induced in response to 7KCh in mRPE cells. (**A**) Western blot estimation of GRP78 levels in mRPE cells exposed to increasing concentrations of 7KCh for 24 h. GRP78 levels were normalized with respect to Actin band quantification. Data are represented as mean ±SEM of at least three different experiments. The dashed line is a guidance of the control value. ANOVA test was used for statistical analysis followed by Tukey post hoc test. (**B**) *XBP1* splicing detection in the RNAseq analysis in mRPE cells treated with 15 μM 7KCh and/or 10 μM SA. Distribution of reads on *XBP1* was visualized using the software IGV (Integrated Genome Viewer). Black arrows indicate the position in which the splicing was performed.

To verify UPR activation, we first evaluated by Western blot the effect on GRP78 accumulation in mRPE cells exposed for 24 h to different concentrations of 7KCh (10–20 μM) (Figure 6A). However, no difference was found between the GRP78 levels detected in control cells and 7KCh-treated cells at any of the doses used. Secondly, since IRE1 promotes XBP1 mRNA splicing and, consequently, its activation [42], we checked whether XBP1 splicing occurs in cells exposed to 7KCh. By using the IGV (Integrative Genome Viewer) software, we found in the mRPE RNAseq samples that exposure to 7KCh induced XBP1 splicing, thus promoting UPR signaling (Figure 6B). Interestingly, treatment with 10 μM SA counteracted XBP1 activation, preventing its splicing.

### 2.7. Both JNK and p38 Pathways Are Involved in the Inflammatory Response to 7KCh in mRPE Cells, but Only p38 Participates in the Cell Death Response

**Figure 7:**
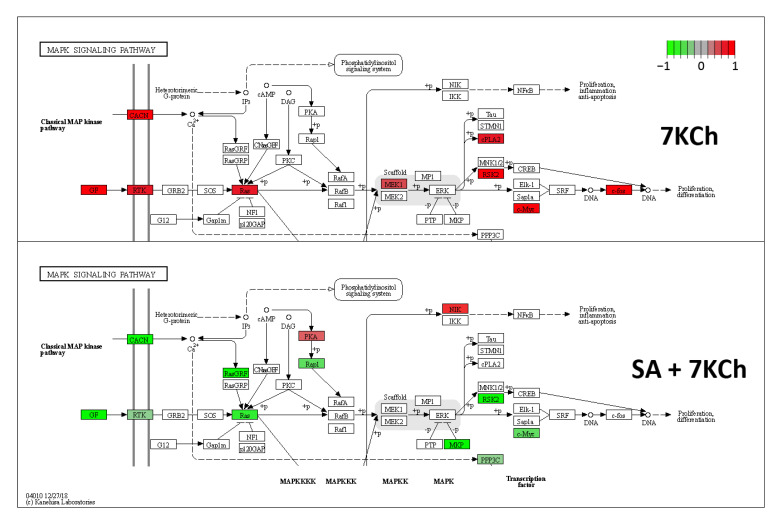
Classical MAPK signaling pathway altered by 15 μM 7KCh treatment in mRPE cells and reversion effect exerted by 10 μM SA over this modulation. In red, proteins expressed at higher levels than control. In green, proteins expressed at lower levels than control. Reprinted and modified with permission from Kyoto Encyclopedia of Genes and Genomes (KEGG). Copyright 2013, KEGG, Kyoto, Japan.

Upregulation of the classical MAPK pathway was observed in response to 7KCh in transcriptomic data, which was almost reversed with co-treatment with SA (Figure 7). The signaling pathways of JNK and p38 are related to MAPK activation. Although at the transcriptomic level no modulation over JNK or p38 was observed in mRPE cells exposed to 15 μM 7KCh, we decided to deepen our investigation into their involvement in the response to 7KCh since these proteins are activated by phosphorylation.

**Figure 8:**
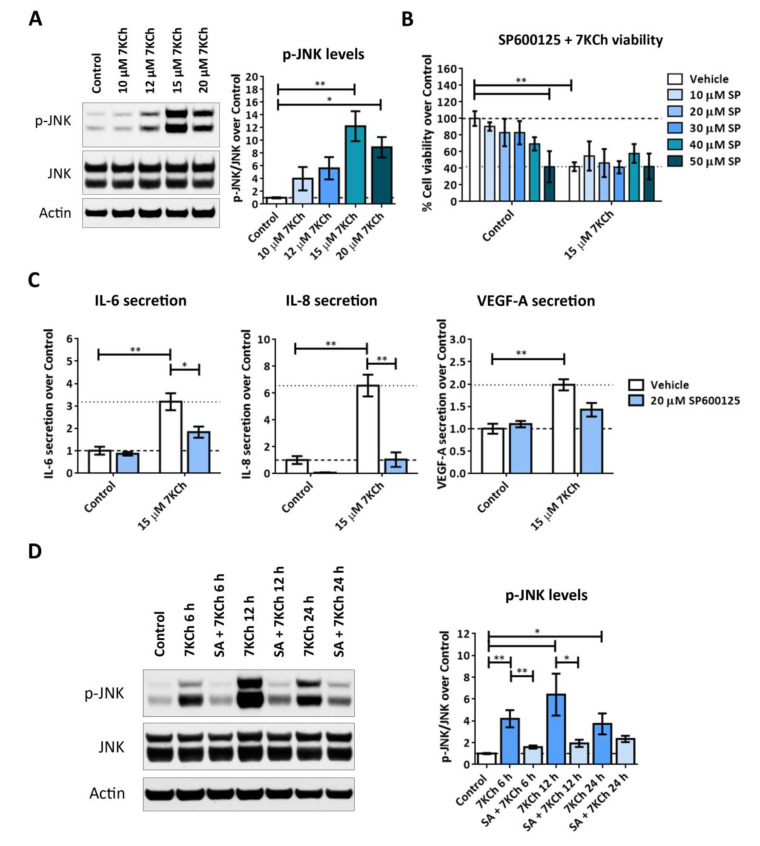
Activation of JNK in response to 7KCh in mRPE cells. (**A**) Western blot estimation of phosphorylated JNK (p-JNK) levels in mRPE cells exposed to increasing concentrations of 7KCh for 24 h. (**B**) Cell viability determined by MTS assay in mRPE cells treated with 15 μM 7KCh and increasing concentrations of SP600125 (SP) for 24 h. (**C**) Secreted levels of IL-6, IL-8 and VEGF-A in mRPE cells exposed to 15 μM 7KCh and 40 μM SP600125 for 48 h and measured with ELISA. (**D**) Western blot estimation of p-JNK levels in mRPE cells exposed to 20 μM 7KCh and 10 μM SA for 6 h, 12 h and 24 h. In Western blot assays, JNK levels, previously normalized with respect to Actin, were used to normalize p-JNK quantification. In MTS assay and ELISA, the JNK inhibitor SP600125 was added with a pretreatment of 2 h with respect to 7KCh. The vehicle group in the graphs represents the control (control-vehicle) and the 7KCh (7KCh-vehicle) treatment. Data are represented as mean ± SEM of at least three different experiments. The dashed and dotted lines are a guidance mark of control and 7KCh value, respectively. ANOVA test was used for statistical analysis followed by Tukey (in **A**,**B**,**D**) or Sidak (in **C**) post hoc test.* *p* < 0.05; ** *p* < 0.01.

We checked by Western blot the JNK phosphorylation in mRPE cells treated with increasing concentrations of 7KCh for 24 h (Figure 8A). A dose-dependent activation was observed, reaching its highest level of phosphorylation with 15 μM 7KCh, and decreasing with 20 μM 7KCh, coinciding with an elevated toxicity of 7KCh (Figure 1A). This suggests a role of JNK in the inflammatory response but not in 7KCh-induced cell death. This observation was confirmed when mRPE cells were treated with 7KCh and SP600125, a JNK-specific inhibitor. SP600125 had no effect on 7KCh-induced toxicity (Figure 8B) but attenuated IL-6 and IL-8 secretion into the medium induced by 7KCh (Figure 8C). To further study JNK activation, we assessed its phosphorylation levels by Western blot at different times in mRPE cells treated with 20 μM 7KCh alone or with 10 μM SA. JNK activation started after 6 h of 7KCh treatment, reaching its maximum level of phosphorylation at 12 h and decreasing at 24 h (Figure 8D), again showing a decrease in JNK activation when 7KCh-induced toxicity is elevated. Co-treatment with 10 μM SA significantly attenuated JNK phosphorylation at both 6 h and 12 h of treatment.

**Figure 9:**
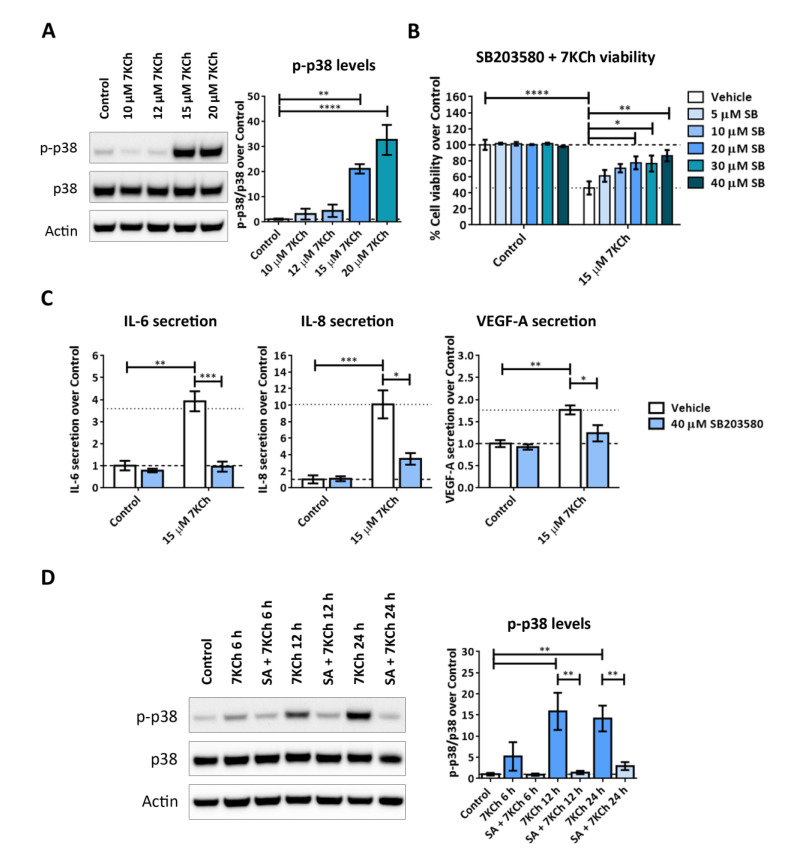
Activation of p38 in response to 7KCh in mRPE cells. (**A**) Western blot estimation of phosphorylated p38 (p-p38) levels in mRPE cells exposed to increasing concentrations of 7KCh for 24 h. (**B**) Cell viability determined by MTS assay in mRPE cells treated with 15 μM 7KCh and increasing concentrations of SB203580 (SB) for 24 h. (**C**) Secreted levels of IL-6, IL-8 and VEGF-A in mRPE cells exposed to 15 μM 7KCh and 40 μM SB203580 for 48 h and measured with ELISA. (**D**) Western blot estimation of p-p38 levels in mRPE cells exposed to 20 μM 7KCh and 10 μM SA for 6 h, 12 h and 24 h. In Western blot assays, p38 levels, previously normalized with respect to Actin, were used to normalize p-p38 quantification. In MTS assay and ELISA, the p38 inhibitor SB203580 was added with a pretreatment of 2 h with respect to 7KCh. The vehicle group in the graphs represents the control (control-vehicle) and the 7KCh (7KCh-vehicle) treatment. Data are represented as mean ± SEM of at least three different experiments. The dashed and dotted lines are a guidance mark of control and 7KCh value, respectively. ANOVA test was used for statistical analysis followed by Tukey (in **A**,**B**,**D**) or Sidak (in **C**) post hoc test.* *p* < 0.05; ** *p* < 0.01; *** *p* < 0.001; **** *p* < 0.0001.

Regarding p38 phosphorylation (p-p38), its activation was observed at 15 μM 7KCh and reached its highest level at a concentration of 20 μM. This suggests that activation of p38 may be related to both 7KCh-induced inflammation and cell death in mRPE cells. The inhibition of p38 with SB203580 significantly reduced the toxicity induced by 15 μM 7KCh in the range of concentrations from 20–40 μM (Figure 9B). In addition, 40 μM SB203580 attenuated the release of the cytokines IL-6, IL-8 and VEGF-A to the medium, promoted by 15 μM 7KCh (Figure 9C). When p38 activation was evaluated in mRPE cells treated with 20 μM 7KCh and 10 μM SA at different times, phosphorylation of p38 was observed after 12 h of treatment and its activation was maintained after 24 h of exposure to 20 μM 7KCh (Figure 9D). On the other hand, co-treatment with 10 μM SA significantly attenuated p38 activation at both 12 h and 24 h. Taken together, these results suggest that both JNK and p38 are involved in the inflammatory response, but only p38 mediates 7KCh-induced cell death.

## 3. Discussion

In this work, we have shown that exposure to 7KCh in RPE cells transcriptionally modulates the expression of several genes associated with different cell processes. Likewise, co-treatment with SA significantly reverses the alteration exerted by 7KCh over several key genes, as well as attenuates the activation of the signaling pathways associated with these genes, highlighting the role of SA as a functional antagonist of this oxysterol in the retina. 

First, we verified the ability of 7KCh to induce both inflammation and cell death in mRPE cells and the capacity of SA to counteract these effects (Figure 1). We had already demonstrated that SA not only protected mRPE cells but also ARPE-19 (human RPE cells) and RF/6A (monkey choroid cells) from 7KCh toxicity [39]. These results are also in line with those previously described in ARPE-19 cells [19,20]. Then, in order to delve into the mechanism of action of 7KCh in the retina, we performed for the first time a genome-wide transcriptome analysis in mRPE cells treated with 15 μM 7KCh. This concentration of 7KCh was selected on the basis that at 15 μM 7KCh, viability has been approximately halved (Figure 1A), but there is still a strong inflammatory induction observed through cytokine release into the medium (Figure 1D). Transcriptome analysis revealed that 15 μM 7KCh upregulates several genes associated with ER stress (UPR signaling), inflammation (cytokines, NFκB and MAPK pathways) and cell death, among others (Figure 2, Table 1 and Table S1). In previous works, authors showed by means of qRT-PCR that 7KCh could increase expression levels of different cytokines and UPR-related genes in ARPE-19 cells [19,20,24,43]. Our results not only validate these previous observations but also offer a broader view of the regulation that 7KCh exerts on retinal cells. On the other hand, we also performed genome-wide transcriptome analysis in mRPE cells treated with both 15 μM 7KCh and 10 μM SA for 24 h. Addition of 10 μM SA significantly reversed the 7KCh-induced modulation over several of these important genes (Figure 3, Table S2).

It is striking the negative regulation that 7KCh provokes on lipid and cholesterol biosynthesis (Table 1). Since oxysterols are formed from cholesterol [44], an upregulation of sterol synthesis would be expected. However, this inhibition of cholesterol synthesis has already been described with other oxysterols, such as 25-hydroxycholesterol [45,46]. In addition, 7KCh treatment in murine oligodendrocyte cells significantly reduced the cellular levels of several sterol and cholesterol precursors [21]. On the other hand, an increase in the expression levels of genes associated with cholesterol transport, such as *ABCA1* or *ABCG1*, is observed in response to 7KCh. These genes are transcriptionally regulated by LXR (liver X receptor), which is activated by oxysterol-binding [45]. The role of LXR in the 7KCh response is controversial; whereas some authors showed that its activation counteracts 7KCh signaling [47,48,49], others demonstrated that LXR mediates cytokine release and cell death induced by this oxysterol [15,50]. A possible explanation of our results is that the cell mistakenly reacts to the presence of oxysterols as an excess of cholesterol and, in response, sterol biosynthesis is repressed, and cholesterol transport is favored. In our previous work, we showed that 10 μM SA negatively regulated lipid and sterol biosynthesis-associated genes in mRPE cells. We also demonstrated that SA modulated the 7KCh response independently of the inhibition of SCD1 and hypothesized that this modulation might be carried out through sterol metabolism [39]. Our current work, however, contradicts this first observation since 7KCh itself represses sterol metabolism. Nevertheless, it validates the fact that the effect of SA over 7KCh is independent of SCD1 inhibition, as 7KCh also reduces *SCD1* expression (Table 1). This is in agreement with a recent study, where SA protected ARPE-19 cells from fenretinide independently of SCD1 inhibition [51]. Regarding genes whose alteration is reversed by adding SA, no effect was found on lipid and sterol metabolism, as would be expected, with the exception of genes activated by LXR and associated with cholesterol transport, such as *ABCA1* and *ABCG1* (Figure 3, Table S2). This suggests that in mRPE cells, perhaps, LXR is important in the induction of the 7KCh response, and that SA exerts part of its inhibition through the repression of this receptor, although further research is needed. 

Next, we evaluated the involvement of different signaling pathways in the response to 7KCh, taking into account data obtained from the transcriptome analysis. TLR4 has been described as the main receptor mediating the 7KCh mechanism of action in ARPE-19 and other cell types [20,52]. Treatment with 15 μM 7KCh increased *TLR4* expression in mRPE cells, and this was attenuated by the addition of 10 μM SA. However, TLR4 inhibition with 10 μM CLI-095 failed to protect mRPE cells from 7KCh-induced toxicity and only significantly decreased IL-6 release to the medium (Figure 4). Thus, our results contradict previous works [20,52] and show minor participation of TLR4 in the response to 7KCh in mRPE cells, although further research is needed to validate these results. 

Then, we assessed the induction of ER stress through UPR signaling, as several genes related to this response were upregulated by 7KCh treatment (Table 1), such as *ATF3*, ASNS, *CEBPG, EIF2S2*, *GFPT1*, *ERN1*, *HSPA5*, *HYOU1*, *SRPRB* and *SSR1*. Since ER stress is favored by increasing levels of ROS [42,53] and there is a wide range of evidence that 7KCh promotes ROS production in different cell types [21,25,27,28,54,55], we first checked ROS release in mRPE cells treated at different times and concentrations of 7KCh. The incubation with 7KCh did not affect ROS release (Figure 5), in disagreement with these studies but in agreement with another study where exposure to 7KCh did not increase ROS production in RPE cells [43]. We also tested the effect of 7KCh treatment on GRP78 accumulation and *XBP1* splicing, two key regulators of UPR signaling [42,56]. Under ER stress conditions, GRP78 binds to unfolded proteins and activates ATF6, IRE1 and PERK, three ER membrane-located receptors that initiate UPR signaling. Once activated, IRE1 promotes the splicing in the *XBP1* mRNA of an intron of 26 nucleotides located between the positions 531–556. The resulting protein enters the nucleus and induces the transcription of several genes involved in ER-associated protein degradation and UPR signaling [42]. We did not see any change in GRP78 protein levels (Figure 6A), contrary to previous works carried out in ARPE-19 cells [19,20]. However, the splicing of *XBP1* was observed in mRPE cells treated with 15 μM 7KCh (Figure 6B), which means that UPR signaling is activated in response to 7KCh in retinal cells. Co-treatment with 10 μM SA successfully avoided *XBP1* splicing. 

Finally, we investigated the implication of MAPK-associated pathways in the 7KCh-induced response in mRPE cells. To begin with, we observed in data obtained from transcriptome sequencing analysis a clear induction of the MAPK classical pathway in response to 15 μM 7KCh, which was attenuated by the addition of 10 μM SA (Figure 7). This contradicts the results obtained by Huang et al. in ARPE-19 cells [20] but is consistent with other studies that show MEK/ERK activation in response to 7KCh, and a protective effect on the inflammation induced by this oxysterol in ARPE-19 cells [24,43] and other cell types [57]. JNK and p38 are two MAPK-related pathways that mediate inflammation and, when their activation persists, can induce cell death [58,59]. Transcriptomic analysis did not show any evidence of activation of either of these two pathways (Table 1 and Table 1 and Table S1). Nevertheless, as these proteins are activated by phosphorylation, we decided to check both JNK and p38 phosphorylation in response to 7KCh. JNK phosphorylation was detected in mRPE cells treated for 24 h with increasing concentrations of 7KCh. However, a decrease in its activation levels was observed with 20 μM 7KCh (Figure 8A), a concentration at which cell death is already well established (Figure 1A). Treatment with the JNK inhibitor SP600125 counteracted cytokine secretion, but had no effect on 7KCh-induced toxicity (Figure 8B,C), indicating a role of JNK only in the inflammatory response to 7KCh. By contrast, activation of p38 was observed in mRPE cells exposed for 24 h to 15 μM and 20 μM 7KCh, and its inhibition with SB203580 attenuated both 7KCh-induced cell death and inflammation (Figure 9A–C). 

Differences between JNK and p38 activation were most evident in mRPE cell samples treated with 20 μM 7KCh at different times (Figure 8D and Figure 9D). JNK phosphorylation was observed after 6 h of treatment with 7KCh, and an important decrease in its activation was detected at 24 h (Figure 8D). On the other hand, p38 activation began after 12 h of treatment, when cell death had not yet been completely triggered, and its phosphorylation levels were maintained after 24 h of exposure to the oxysterol (Figure 9D). These results suggest that in RPE cells, JNK is activated before p38, and both signaling pathways are involved in the inflammatory response to 7KCh, but only p38 participates in the toxic response. These data could also indicate that p38 may trigger the switch between inflammatory and cell death responses. The activation of JNK and p38 has already been reported in response to 7KCh-induced inflammation in different cell types, including ARPE-19 cells [43,54,57,60,61]. Recently, p38 has also been associated with 7KCh modulation of cell adhesion in leukocytes [23]. However, to our knowledge, there are no previous studies demonstrating a direct implication between p38 activation and 7KCh-induced cell death. Once again, co-treatment with 10 μM SA significantly reduced p-JNK levels after 6 h and 12 h of treatment, and p-p38 levels after 12 h and 24 h of treatment, highlighting the potential, once again, of this molecule counteracting 7KCh effects in these cells (Figure 8D and Figure 9D).

The relationship between 7KCh and the development of AMD is well established since this oxysterol is one of the main molecules accumulated in drusen [4,19,20,26,29,30,31,32,33,34,39,43]. ER stress activation and alterations in MAPK signaling have been associated with the pathogenesis of AMD [62,63,64,65]. In this study, we have demonstrated that 7KCh induces these responses in RPE cells; in addition, we have provided a broader view of the alterations that this oxysterol causes at the transcriptomic level. Thus, the characterization of the 7KCh-induced response in retinal cells offers new opportunities to search for alternative therapeutic targets for AMD. Likewise, SA is, currently, the molecule that has worked best counteracting the harmful effect of 7KCh on retinal cells. The potential of SA as a treatment for AMD has already been revealed due to its ability to attenuate the 7KCh response in ARPE-19 cells, as well as the deleterious effect of fenretinide in this cell line [19,20,51]. In our previous study, we highlighted the benefits of SA administration in three retinal cell lines and its potential in the treatment of ocular diseases [39]. In our current work, we have demonstrated that SA attenuates the response to 7KCh in mRPE cells at different levels, as well as its ability to reverse part of the gene modulation that 7KCh exposure exerts on these cells, providing new evidence of SA therapeutic capacity. Although there is still a long way to go and further research is needed, SA is presented as a new opportunity to improve or complement therapies currently used to treat AMD. 

## 4. Materials and Methods

### 4.1. Cell Lines and Culture

Monkey retinal pigment epithelium (mRPE) cells were obtained as a gift from Dr. SP Becerra from the National Eye Institute (NIH, Bethesda, MD, USA) and derived from 3- to 5-year-old Rhesus monkey eyes. Once obtained, mRPE cells were kept in a stable monolayer for two weeks until the expression of biochemical and physiological markers of differentiated tissue (pigmentation, polarization RPE65 expression) was achieved [66]. Cells were grown in DMEM/F12 1:1 medium (Hyclone-Thermo Scientific, Waltham, MA, USA) supplemented with 5% fetal bovine serum (Invitrogen, Alcobendas, Madrid, Spain), 1.5% pyruvate, 1% non-essential amino-acids and 1% penicillin/streptomycin (Hyclone-Thermo Scientific, Waltham, MA, USA). Cultured cells were maintained in an atmosphere of 37 °C containing 5% CO_2_ and 85% humidity.

### 4.2. Cell Treatments

Cells were seeded in 12-well plates for cell viability or in P100 plates (Corning, New York, NY, USA) for RNA and protein purification at a density of 100.000 cells/well or 1.2 × 10^6^ cells/plate, respectively. Then, cells were allowed to attach and reach 100% confluency for 24 h before removing the medium and replacing it with serum-free medium for 24 h. Treatments were then added and maintained for 24 h, with the exception of ELISAs that were maintained for 48 h, and some protein extracts that were collected at shorter times. Cells were treated with 8–20 μM 7KCh (Sigma-Aldrich, Madrid, Spain) alone or with 10 μM SA (PPQF, University of Alcalá, Madrid, Spain), 10 μM of the TLR4 inhibitor CLI-095 (Invivogen Inc., San Diego, CA, USA), 10–50 μM of the JNK inhibitor SP600125 (StressMarq Biosciences Inc., Victoria, BC, Canada) or 5–40 μM of the p38 inhibitor SB203580 (Sigma-Aldrich, Madrid, Spain). 7KCh was prepared in β-cyclodextrin (Sigma-Aldrich, Madrid, Spain) as previously described [15,20], CLI-095 was dissolved in culture medium and SA, SP600125 and SB203580 were dissolved in DMSO (Sigma-Aldrich, Madrid, Spain). 

### 4.3. Cell Viability Assays

Cell toxicity or protective effect of the different treatments was validated using CellTitter 96 Aqueous One Solution Reagent MTS assay (Promega, Madison, WI, USA). This assay measures cell metabolic activity by the reduction of the MTS compound (3-(4,5-dimethylthiazol-2-yl)-5-(3-carboxymethoxyphenyl)-2-(4-sulfophenyl)-2H-tetrazolium), a reaction that is only carried out by living cells. Cells were washed two times with PBS before adding the MTS reagent at a concentration of 1:10 in culture medium. A baseline measure of the absorbance at 490 nm was performed using Biotek Synergy H4 multi-mode plate reader (BioTek Instruments, Covina, CA, USA) and then a final measurement was performed after 4 h of incubation at 37 °C. Results were presented as percentage viability with respect to control vehicle cells. 

### 4.4. ROS Detection Assay

The release of ROS was determined by measuring the fluorescence resulting from the oxidation of the probe H_2_DCFDA (2,7-dichlorodihydrofluorescein diacetate, Invitrogen, Madrid, Spain) into DFC (dichlorofluorescein), as this oxidation is promoted by ROS. Cells were incubated with 10 μM H_2_DCFDA freshly prepared in 1× PBS after 8 h or 24 h of treatment. Then, fluorescence was measured using Biotek Synergy H4 multi-mode plate reader (BioTek Instruments, Covina, CA, USA) with 488 nm excitation and 535 nm emission filters. 

### 4.5. RNA Purification

Total RNA was isolated from cell cultures in 1 mL of TRIzol (Invitrogen, Madrid, Spain). Then, the RNA was purified with the RNeasy mini-kit (Qiagen, Valencia, CA, USA) and DNase I (Qiagen, Valencia, CA, USA) according to manufacturer’s guidelines.

### 4.6. Quantitative Real-Time PCR

Reverse transcription was performed from 1 μg of total RNA with random primers and the SuperScript III kit (Invitrogen, Madrid, Spain) in order to synthesize first-strand DNA following manufacturer’s instructions. Then, the cDNA obtained was mixed with 0.3 μM forward and reverse oligonucleotide primers (Table 2) and SYBR Green PCR Master Mix (Applied Biosystems, Carlsbad, CA, USA) to carry out real-time polymerase chain reaction (qRT-PCR). Quantitative measures were taken using a 7300 Real-Time PCR System (Applied Biosystems, Madrid, Spain). Cycling conditions were: an initial denaturation at 95 °C for 10 min, followed by 40 cycles of 95 °C for 15 s and 60 °C for 1 min. Finally, to validate amplicon specificity, the dissociation curve was implemented from 60 °C to 95 °C. Gene expression levels were calculated by interpolating Ct value (threshold cycle) from the corresponding standard line and using *18S* rRNA expression to normalize the results. 

### 4.7. Next-Generation Sequencing

Ultrasequencing was carried out using Illumina reagents (Illumina, San Diego, CA, USA) and protocols as previously described [67]. The integrity and quality of total RNA was assessed with the Experion™ automated electrophoresis system (Bio-Rad, Hercules, CA, USA). Next, mRNA was isolated from 1 μg of total RNA with Illumina’s Ribo-zero plus rRNA depletion kit (Illumina, Madrid, Spain), and cleaved into approximately 200 bp fragments by divalent cations at high temperatures. The first cDNA strand was obtained by reverse transcription of the mRNA fragments with random primers and reverse transcriptase, whereas the second cDNA strand was synthesized using DNA polymerase I and RNase H. The cDNA double-stranded fragments were end-repaired using T4 DNA polymerase and Klenow DNA polymerase, then phosphorylated by T4 polynucleotide kinase and, finally, ligated to Illumina Indexed Adapters. The resulting adapter-tagged libraries were amplified with 15 PCR cycles and using DNA polymerase (Phusion, Finnzymes Reagents, Vantaa, Finland). Validation and quantification were performed using the Experion™ automated electrophoresis system and qPCR, respectively. Then, pools of six indexed libraries were mixed at equimolar ratios to reach a total oligonucleotide concentration of 10 mM. Finally, libraries obtained were sequenced on the Illumina Genome Analyzer IIx platform in order to generate 150 bp single reads, sequencing six groups of libraries per lane.

Raw sequence data in fastq format were cleaned of low-quality and adapter sequencing. Reads were then mapped using the aligner STAR (https://github.com/alexdobin/STAR (version 2.7.8a, accessed on 20 February 2021) to the reference genome of *Macaca mulatta* (Mmul_8.0.1) and counted with FeatureCounts (http://subread.sourceforge.net/ (accessed on 1 April 2021)). Normalization and global distance analysis of reads were performed with DESeq2 and the differential expression analysis was carried out using the R package [68]. The analysis of altered pathways promoted by the treatment of 7KCh alone or with the combination of SA was performed by studying significant up- and downregulated genes from high-throughput sequencing. Specialized software (www.genemania.org, www.reactome.org, accessed on 15 December 2022) was used to find cellular pathways differentially expressed and shown in the Kyoto Encyclopedia of Genes and Genomes (KEGG) [69,70,71]. The software IGV was used to visualize the splicing of *XBP1* [72].

### 4.8. Western Blotting

Protein extracts were obtained from scraped cells in culture using RIPA buffer (Thermo Scientific, Madrid, Spain) containing phosphatase (PhosStop, Roche, Basilea, Switzerland) and protease (EDTA-free complete, Roche, Basilea, Switzerland) inhibitors. Supernatants were collected from homogenates by centrifugation for 30 min at 15,000× *g*, and protein concentration was determined using a BCA kit (Pierce, Rockford, IL, USA) following manufacturer’s guidelines and using Biotek Synergy H4 multi-mode plate reader (BioTek Instruments, Covina, CA, USA) to measure the absorbance at 562 nm. Next, 20 μg of sample mixed with 10× sample reducing agent (Invitrogen, Madrid, Spain) and 4× sample Buffer (Invitrogen, Madrid, Spain) were heated for 10 min at 70 °C. SeeBlue plus 2 pre-stained standard (Invitrogen, Madrid, Spain) was used as a molecular weight marker and was run with the samples on 4–12% SDS–polyacrylamide gels (Invitrogen, Madrid, Spain). After that, proteins were transferred onto 0.2 μm polyvinylidene difluoride (PVDF) membranes (iBlot system, Invitrogen, Madrid, Spain). Blocking was performed for 2 h at room temperature with 5% non-fat dry milk dissolved in 1× TBS (tris-buffered saline) and 0.1% Tween (Sigma-Aldrich, Madrid, Spain).

Membranes were then incubated overnight at 4 °C with different primary antibodies for protein identification: α-GRP78 1:1000 (#3183, Cell Signaling, Danvers, MA, USA), α-p-JNK 1:1000 (ab124956, Abcam, Cambridge, UK), α-JNK 1:5000 (ab179461, Abcam, Cambridge, UK), α-p-p38 1:5000 (#4511, Cell Signaling, Danvers, MA, USA) or α-p38 1:5000 (#8690, Cell Signaling, Danvers, MA, USA). Protein levels were standardized with the detection of α-Actin at a dilution of 1:10000 (#A5441, Sigma-Aldrich, Madrid, Spain) in the same membranes. Immunoreactivity was demonstrated by incubating the membranes with α-mouse (715-035-1514, Jackson Immunoresearch Lab. West Grobe, PA, USA) or α-rabbit (7074, Cell Signaling) and using the chemiluminescence kit ClarityTM Western ECL (BIO-RAD, Berkeley, CA, USA), and exposing to X-ray films (Amersham Hyperfilm ECL, GE Healthcare, Buckinghamshire, UK) or using the ChemiDoc Imaging System (Bio-Rad, Hercules, CA, USA).

### 4.9. ELISAs

Levels of secreted IL-6, IL-8 and VEGF-A were measured in conditioned medium of mRPE cells treated with 15 μM 7KCh and/or 10 μM SA, 10 μM CLI-095 or 40 μM SB203580 for 48 h using Macaque IL-6 ELISA kit (Raybiotech Life, Peachtree Corners, GA, USA), Macaque IL-8 ELISA kit (Raybiotech Life, Peachtree Corners, GA, USA) and Monkey Vascular Endothelial Growth Factor A ELISA kit (MyBiosource, San Diego, CA, USA), respectively. The culture medium was collected following manufacturer’s instructions and absorbance was measured at 450 nm using the Biotek Synergy H4 multi-mode plate reader (BioTek Instruments, Covina, CA, USA).

### 4.10. Statistical Analysis

Statistical analysis was performed using GraphPad Prism 6 software. Data were expressed as means ± SEM (standard error of the means) and were considered statistically significant when *p* ≤ 0.05. ANOVA test was used to analyze normally distributed data followed by Sidak or Tukey post hoc test, following the program’s recommendation.

## Figures and Tables

**Table 1:** Relevant genes and associated pathways altered in mRPE cells treated with 15 μM 7KCh for 24 h. Positive Log2FC (FC, Fold Change) represents upregulated genes, while negative Log2FC represents downregulated genes.

Pathway	Gene ID	Log2FC	FDR	Gene Description
Lipid metabolism	*ACACA*	−0.69	4.43 × 10^−5^	acetyl-CoA carboxylase 1
	*ABHD5*	0.66	2.64 × 10^−2^	abhydrolase domain containing 5
	*ACLY*	−0.76	7.19 × 10^−8^	ATP-citrate synthase
	*CAV1*	−0.53	4.64 × 10^−2^	Caveolin-1
	*ELOVL4*	0.99	2.57 × 10^−2^	Elongation very long chain fatty acids protein 4
	*FADS1*	−0.82	1.86 × 10^−5^	fatty acid desaturase 1
	*FADS2*	−1.02	1.05 × 10^−7^	fatty acid desaturase 2
	*FASN*	−1.17	2.21 × 10^−8^	Fatty acid synthase
	*SCD*	−0.93	2.26 × 10^−7^	Stearoyl-CoA desaturase
Sterol biosynthesis and transport	*ABCA1*	1.83	1.91 × 10^−14^	ATP binding cassette subfamily A member 1
	*ABCG1*	2.95	9.60 × 10^−21^	ATP binding cassette subfamily G member 1
	*ABCC9*	2.48	6.32 × 10^−5^	ATP binding cassette subfamily C member 9
	*FDFT1*	−1.45	1.38 × 10^−26^	squalene synthase
	*HMGCR*	−1.65	3.23 × 10^−17^	3-hydroxy-3-methylglutaryl coenzyme A reductase
	*HMGCS1*	−3.1	2.56 × 10^−51^	hydroxymethylglutaryl-CoA synthase
	*INSIG1*	−2.09	9.80 × 10^−28^	insulin-induced gene protein
	*LDLR*	−0.8	4.65 × 10^−3^	low-density lipoprotein receptor
	*LSS*	−2.08	6.49 × 10^−44^	terpene cyclase/mutase family member
	*MSMO1*	−2.2	2.28 × 10^−42^	methylsterol monooxygenase 1
	*MVD*	−2.4	4.33 × 10^−37^	diphosphomevalonate decarboxylase
	*MVK*	−1.13	4.66 × 10^−8^	mevalonate kinase
	*SQLE*	−2.11	1.36 × 10^−44^	squalene monooxygenase
ER stress	*ASNS*	1.88	3.62 × 10^−7^	glutamine-dependent asparagine synthetase
	*ATF3*	3.16	1.58 × 10^−8^	activating transcription factor 3
	*CEBPB*	1.44	3.54 × 10^−4^	CCAAT/enhancer-binding protein beta
	*CEBPG*	1.09	1.71 × 10^−4^	CCAAT/enhancer-binding protein gamma
	*EIF2S2*	0.46	3.81 × 10^−2^	eukaryotic translation initiation factor 2 subunit beta
	*ERN1*	0.83	1.33 × 10^−3^	non-specific serine/threonine protein kinase
	*GFPT1*	0.91	2.52 × 10^−3^	glutamine--fructose-6-phosphate transaminase 1
	*HSPA5*	1.36	1.94 × 10^−5^	heat Shock Protein Family A (Hsp70) Member 5
	*HYOU1*	0.81	1.40 × 10^−3^	hypoxia up-regulated 1
	*PREB*	0.63	8.42 × 10^−3^	prolactin regulatory element binding
	*SRPRB*	0.82	4.86 × 10^−4^	SRP receptor subunit beta
	*SSR1*	0.47	2.21 × 10^−2^	signal sequence receptor subunit 1
NFκB and MAPK inflammatory signaling	*CXCL2*	1.3	1.99 × 10^−2^	C-X-C motif chemokine 2
	*EGFR*	0.62	2.55 × 10^−2^	receptor protein-tyrosine kinase
	*FOSB*	2.81	2.50 × 10^−2^	FosB proto-oncogene
	*IL1A*	2.85	4.87 × 10^−5^	interleukin-1 alpha
	*IL6*	1.36	4.98 × 10^−2^	interleukin-6
	*MAP2K1*	0.46	1.83 × 10^−2^	mitogen-activated protein kinase kinase 1
	*MAP3K8*	0.89	4.25 × 10^−2^	mitogen-activated protein kinase kinase kinase 8
	*MYC*	0.78	4.90 × 10^−5^	mYC proto-oncogene
	*NFKBIA*	0.55	1.83 × 10^−2^	NF-kappa-B inhibitor alpha
	*NFKBIB*	1.03	7.77 × 10^−5^	NF-kappa-B inhibitor beta
	*NFKBIE* *RPS6KA1*	0.540.95	4.83 × 10^−2^2.22 × 10^−3^	NF-kappa-B inhibitor epsilonribosomal Protein S6 Kinase A1
	*RRAS2*	0.74	1.14 × 10^−3^	Ras related 2
	*TLR4*	0.96	2.31 × 10^−3^	toll-like receptor 4
	*TRAF1*	0.82	2.35 × 10^−2^	TNF Receptor Associated Factor 1
	*VEGFA*	0.74	3.42 × 10^−2^	vascular endothelial growth factor A
Cell death signaling	*APAF1*	−0.45	3.16 × 10^−2^	apoptotic peptidase activating factor 1
	*CASP1*	1.40	3.21 × 10^−7^	caspase-1
	*DFNA5*	0.95	9.87 × 10^−4^	non-syndromic hearing impairment protein 5 isoform A
	*GADD45A*	0.70	1.99 × 10^−5^	growth arrest and DNA damage-inducible alpha
	*PMAIP1*	1.30	9.07 × 10^−6^	phorbol-12-Myristate-13-Acetate-Induced Protein 1
	*TNFRSF10B*	0.60	2.36 × 10^−2^	TNF receptor superfamily member 10b
	*TP53I3*	0.65	2.57 × 10^−2^	tumor protein p53 inducible protein 3

**Table 2:** Primers used in this work in mRPE cells.

Gene	Oligonucleotide Sequence
*18S*—Forward	5′-ATGCTCTTAGCTGAGTGTCCCG-3′
*18S*—Reverse	5′-ATTCCTAGCTGCGGTATCCAGG-3′
*IL8*—Forward	5′-ACCAAGGAAATCGGCCCCTA-3′
*IL8*—Reverse	5′-CCATACCTCTAGGCTGGCTATC-3′
*IL6*—Forward	5′-CTGGTCTTTTGGAGTTTGAGGT-3′
*IL6*—Reverse	5′-GCTGGCATTTGTGGTTGGT-3′
*VEGFA*—Forward	5′-TTCACCCTCGTCCTCTTCCT-3′
*VEGFA*—Reverse	5′-ATCCTGCCCTGTCTCTCTGT-3′

## Data Availability

Not applicable.

## Supplementary Materials

The following supporting information can be downloaded at: https://www.mdpi.com/article/10.3390/ijms24087459/s1.

## Abbreviations

7KCh	7-Ketocholesterol
AMD	age-related macular degeneration
CNV	choroidal neovascularization
DEG	differentially expressed genes
ER	endoplasmic reticulum
FC	fold change
FDR	false discovery rate
GO	gene ontology
IGV	Integrative Genome Viewer
KEGG	Kyoto Encyclopedia of Genes and Genomes
LXR	liver X receptor
mRPE	monkey retinal pigment epithelium
qRT-PCR	real-time polymerase chain reaction
RPE	retinal pigment epithelium
SA	sterculic acid
SCD1	stearoyl coenzyme-A desaturase 1
SEM	standard error of the means
TBS	tris-buffered saline
UPR	unfolded protein response
