# Supplementary material for: Identification of 7-Ketocholesterol-Modulated Pathways and Sterculic Acid Protective Effect in Retinal Pigmented Epithelium Cells by Using Genome-Wide Transcriptomic Analysis
Source: Int J Mol Sci. 2023 Apr 18;24(8):7459. doi: 10.3390/ijms24087459 (PMC10144535; doi:10.3390/ijms24087459)
Supplement: Supplementary file 1 [file ijms-24-07459-s001.zip › ijms-2327330 supplementary.pdf]

International Journal of
Molecular Sciences

MDPI

Article

Table S1. Complete list of DEGs found between control mRPE cells and treated with 15 µM 7KCh. Positive Log2FC represents upregulated genes while negative Log2FC represents downregulated genes.

Ensemble ID	Gene ID	Log2FC	FDR	Gene description
ENSMMUG00000001226	*HMGCS1*	-3.10	2.56E-51	3-hydroxy-3-methylglutaryl-CoA synthase 1
ENSMMUG00000003535	*SQLE*	-2.11	1.36E-44	squalene epoxidase
ENSMMUG00000022329	*LSS*	-2.08	6.49E-44	lanosterol synthase
ENSMMUG00000039955	*MSMO1*	-2.20	2.28E-42	methylsterol monooxygenase 1
ENSMMUG00000017115	*IDI1*	-2.27	5.27E-40	isopentenyl-diphosphate delta isomerase 1
ENSMMUG00000014326	*MVD*	-2.40	4.33E-37	mevalonate diphosphate decarboxylase
ENSMMUG00000038190	*CYP51A1*	-1.85	4.36E-30	lanosterol 14-alpha demethylase
ENSMMUG00000044056	*EBP*	-1.62	5.87E-28	EBP cholestenol delta-isomerase
ENSMMUG00000047977	*INSIG1*	-2.09	9.80E-28	insulin induced gene 1
ENSMMUG00000019211	*FDFT1*	-1.45	1.38E-26	farnesyl-diphosphate farnesyltransferase 1
ENSMMUG00000018058	*ABCG1*	2.95	9.60E-21	ATP binding cassette subfamily G member 1
ENSMMUG00000021577	*DHCR24*	-1.58	1.58E-20	24-dehydrocholesterol reductase
ENSMMUG00000023144	*FDPS*	-1.25	2.49E-20	farnesyl diphosphate synthase
ENSMMUG00000023089	*PHGDH*	2.90	6.43E-20	phosphoglycerate dehydrogenase
ENSMMUG00000016698	*PNPLA3*	-1.48	1.75E-19	patatin like phospholipase domain containing 3
ENSMMUG00000000006	*NEURL3*	3.72	6.12E-19	neuralized E3 ubiquitin protein ligase 3
ENSMMUG00000017932	*HMGCR*	-1.65	3.23E-17	3-hydroxy-3-methylglutaryl-CoA reductase
ENSMMUG00000006139	*ACSS2*	-1.31	3.23E-17	acyl-CoA synthetase short chain family member 2
ENSMMUG00000011845	*HK2*	1.70	8.68E-17	hexokinase 2
ENSMMUG00000013176	*NSDHL*	-1.18	1.82E-16	NAD(P) dependent steroid dehydrogenase-like
ENSMMUG00000014520	*ITIH1*	-3.68	6.68E-16	inter-alpha-trypsin inhibitor heavy chain 1
ENSMMUG00000011976	*DHCR7*	-1.48	8.29E-15	7-dehydrocholesterol reductase
ENSMMUG00000001618	*ATP6V0D2*	6.06	1.44E-14	ATPase H+ transporting V0 subunit d2
ENSMMUG00000020608	*ABCA1*	1.83	1.91E-14	ATP binding cassette subfamily A member 1
ENSMMUG00000003288	*GPT2*	1.61	9.13E-14	glutamic--pyruvic transaminase 2
ENSMMUG00000018773	*TM7SF2*	-1.30	5.37E-13	transmembrane 7 superfamily member 2
ENSMMUG00000009332	*HSD17B7*	-1.08	7.85E-12	3-keto-steroid reductase
ENSMMUG00000018907	*ERFE*	1.85	1.35E-10	Erythroferrone
ENSMMUG00000011245	*CRISPLD2*	-1.23	2.09E-10	cysteine rich secretory protein LCCL domain containing 2
ENSMMUG00000011868	*PSAT1*	3.00	2.27E-10	phosphoserine aminotransferase 1
ENSMMUG00000039289	*SC5D*	-1.41	4.75E-10	sterol-C5-desaturase
ENSMMUG00000008140	*SGK1*	1.31	1.28E-09	serum/glucocorticoid regulated kinase 1
ENSMMUG00000019653	*SLC7A8*	-1.05	1.69E-09	solute carrier family 7 member 8
ENSMMUG00000005742	*CDCP1*	1.92	5.05E-09	CUB domain containing protein 1
ENSMMUG00000021806	*VGLL4*	-1.00	7.59E-09	vestigial like family member 4
ENSMMUG00000000484	*C2CD4C*	-2.28	1.04E-08	C2 calcium dependent domain containing 4
ENSMMUG00000012185	*ATF3*	3.16	1.58E-08	activating transcription factor 3
ENSMMUG00000003273	*BCAM*	-1.16	1.94E-08	basal cell adhesion molecule
ENSMMUG00000012811	*FASN*	-1.17	2.21E-08	fatty acid synthase
ENSMMUG00000000567	*BAMBI*	-1.01	4.50E-08	MP and activin membrane bound inhibitor
ENSMMUG00000011663	*MVK*	-1.13	4.66E-08	mevalonate kinase
ENSMMUG00000018931	*STARD4*	-1.28	7.19E-08	StAR related lipid transfer domain containing 4
ENSMMUG00000008563	*ACLY*	-0.76	7.19E-08	ATP citrate lyase
ENSMMUG00000004504	*KITLG*	3.19	9.75E-08	KIT ligand

ENSMMUG00000000327	*FADS2*	-1.02	1.05E-07	fatty acid desaturase 2
ENSMMUG00000013610	*ETV5*	1.42	1.33E-07	ETS variant transcription factor 5
ENSMMUG00000009289	*CBS*	1.26	1.76E-07	cystathionine beta-synthase
ENSMMUG00000002605	*UCP2*	-1.00	1.84E-07	uncoupling protein 2
ENSMMUG00000013507	*LAD1*	-1.26	2.26E-07	ladinin 1
ENSMMUG00000010817	*SCD*	-0.93	2.26E-07	stearoyl-CoA desaturase
ENSMMUG00000009852	*CASP1*	1.40	3.21E-07	caspase-1
ENSMMUG00000019157	*CTH*	2.31	3.62E-07	cystathionine gamma-lyase
ENSMMUG00000019326	*ASNS*	1.88	3.62E-07	asparagine synthetase (glutamine-hydrolyzing)
ENSMMUG00000011909	*RBP3*	-1.16	6.97E-07	retinol binding protein 3
ENSMMUG00000041958	*BHLHA15*	3.97	7.70E-07	basic helix-loop-helix family member a15
ENSMMUG00000009022	*GDPD5*	-1.09	8.06E-07	glycerophosphodiester phosphodiesterase domain containing 5
ENSMMUG00000009739	*MTHFD2*	1.41	9.38E-07	methylenetetrahydrofolate dehydrogenase 2, methenyltetrahydrofolate cyclohydrolase
ENSMMUG00000038480	*EIF4EBP1*	1.42	1.26E-06	eukaryotic translation initiation factor 4E binding protein 1
ENSMMUG00000020322	*ALDOC*	-1.09	1.32E-06	Aldolase, fructose-bisphosphate C
ENSMMUG00000005947	*SAMD11*	-1.14	1.53E-06	sterile alpha motif domain containing 11
ENSMMUG00000006269	*CSTA*	2.21	2.31E-06	cystatin A
ENSMMUG00000019996	*ENC1*	-0.92	2.60E-06	ectodermal-neural cortex 1
ENSMMUG00000006971	*FKBP11*	0.99	3.06E-06	FKBP prolyl isomerase 11
ENSMMUG00000007090	*DOK5*	1.22	3.48E-06	docking protein 5
ENSMMUG00000015474	*MYLIP*	0.84	4.52E-06	myosin regulatory light chain interacting protein
ENSMMUG00000044653	*SLC38A10*	-0.87	5.66E-06	solute carrier family 38 member 10
ENSMMUG00000003292	*CLDN4*	2.75	5.66E-06	claudin 4
ENSMMUG00000009084	*TM6SF1*	2.16	7.20E-06	transmembrane 6 superfamily member 1
ENSMMUG00000011593	*RGCC*	3.60	8.50E-06	regulator of cell cycle
ENSMMUG00000001119	*UTS2R*	-2.21	8.53E-06	urotensin 2 receptor
ENSMMUG00000040573	*PMAIP1*	1.30	9.07E-06	phorbol-12-myristate-13-acetate-induced protein 1
ENSMMUG00000017561	*SLC43A2*	-1.31	9.79E-06	solute carrier family 43 member 2
ENSMMUG00000014176	*CYTIP*	1.16	1.03E-05	cytohesin 1 interacting protein
ENSMMUG00000022893	*VWF*	-3.52	1.21E-05	von Willebrand factor
ENSMMUG00000007037	*UPP1*	2.05	1.28E-05	uridine phosphorylase 1
ENSMMUG00000012064	*C7H14orf1*	-0.97	1.66E-05	ergosterol biosynthesis 28 homolog
ENSMMUG00000010927	*TNFAIP6*	2.26	1.85E-05	TNF alpha induced protein 6
ENSMMUG00000013798	*FADS1*	-0.82	1.86E-05	fatty acid desaturase 1
ENSMMUG00000005217	*HSPA5*	1.36	1.94E-05	heat shock protein family A (Hsp70) member 5
ENSMMUG00000014542	*CHAC1*	2.46	1.97E-05	ChaC glutathione specific gamma-glutamylcy-clotransferase 1
ENSMMUG00000038425	*GADD45A*	0.70	1.99E-05	growth arrest and DNA damage inducible alpha
ENSMMUG00000012718	*DPYSL2*	-0.68	2.17E-05	dihydropyrimidinase like 2
ENSMMUG00000010601	*PRG2*	2.24	2.22E-05	proteoglycan 2, pro-eosinophil major basic protein
ENSMMUG00000017169	*FAM107B*	1.23	2.77E-05	family with sequence similarity 107 member B
ENSMMUG00000009223	*PPP1R15A*	1.56	2.77E-05	protein phosphatase 1 regulatory subunit 15A
ENSMMUG00000004771	*CXCL14*	-1.03	3.50E-05	C-X-C motif chemokine ligand 14
ENSMMUG00000019992	*GRB10*	1.64	3.53E-05	growth factor receptor bound protein 10
ENSMMUG00000017327	*SLAMF7*	2.79	3.60E-05	SLAM family member 7
ENSMMUG00000017916	*LIPE*	-0.91	3.88E-05	lipase E, hormone sensitive type
ENSMMUG00000009349	*ACACA*	-0.69	4.43E-05	acetyl-CoA carboxylase alpha
ENSMMUG00000019777	*IL1A*	2.85	4.87E-05	interleukin 1 alpha
ENSMMUG00000016230	*TMEM63B*	-0.73	4.88E-05	transmembrane protein 63B
ENSMMUG00000041773	*MYC*	0.78	4.90E-05	MYC proto-oncogene, bHLH transcription factor
ENSMMUG00000014601	*FABP5*	0.80	4.90E-05	fatty acid binding protein 5

ENSMMUG00000017470	*THRSP*	-2.46	5.41E-05	thyroid hormone responsive
ENSMMUG00000007027	*ACTC1*	-1.55	5.62E-05	actin alpha cardiac muscle 1
ENSMMUG00000020806	*ABCC9*	2.48	6.32E-05	ATP binding cassette subfamily C member 9
ENSMMUG00000023801	*LCN2*	4.72	6.55E-05	lipocalin 2
ENSMMUG00000023643	*NFKBIB*	1.03	7.77E-05	NFKB inhibitor beta
ENSMMUG00000015851	*SFRP2*	-0.69	8.87E-05	secreted frizzled related protein 2
ENSMMUG00000028717	*PTPRH*	1.22	9.02E-05	protein tyrosine phosphatase receptor type H
ENSMMUG00000003874	*CTSD*	-0.71	9.28E-05	cathepsin D
ENSMMUG00000004628	*IARS*	0.72	9.60E-05	isoleucyl-tRNA synthetase 1
ENSMMUG00000015314	*TSPAN15*	-0.66	1.08E-04	tetraspanin 15
ENSMMUG00000013589	*ACBD4*	-0.74	1.31E-04	acyl-CoA binding domain containing 4
ENSMMUG00000001048	*EAF2*	1.11	1.44E-04	ELL associated factor 2
ENSMMUG00000013407	*ADAMTS1*	0.78	1.65E-04	ADAM metallopeptidase with thrombospondin type 1 motif 1
ENSMMUG00000047896	*TP53INP1*	-0.80	1.69E-04	tumor protein p53 inducible nuclear protein 1
ENSMMUG00000007659	*XPOT*	0.77	1.71E-04	exportin for Trna
ENSMMUG00000006127	*CEBPG*	1.09	1.71E-04	CCAAT enhancer binding protein gamma
ENSMMUG00000015001	*LIMK2*	-0.72	1.72E-04	LIM domain kinase 2
ENSMMUG00000004719	*GARS*	1.02	1.75E-04	glycyl-tRNA synthetase 1
ENSMMUG00000004706	*TBL2*	0.85	1.75E-04	transducin beta like 2
ENSMMUG00000042454	*PHLDA1*	1.15	1.75E-04	pleckstrin homology like domain family A member 1
ENSMMUG00000009093	*IGSF9*	-0.80	1.75E-04	immunoglobulin superfamily member 9
ENSMMUG00000021120	*MAGED1*	-0.80	1.83E-04	MAGE family member D1
ENSMMUG00000010189	*TMEM71*	2.87	1.83E-04	transmembrane protein 71
ENSMMUG00000005618	*TNNC1*	-0.79	1.83E-04	troponin C1, slow skeletal and cardiac type
ENSMMUG00000010164	*VGF*	1.71	1.88E-04	VGF nerve growth factor inducible
ENSMMUG00000019945	*FGFR3*	-1.18	1.95E-04	fibroblast growth factor receptor 3
ENSMMUG00000043586	*ID3*	-1.05	1.97E-04	inhibitor of DNA binding 3
ENSMMUG00000040085	*SLC33A1*	0.79	1.99E-04	solute carrier family 33 member 1
ENSMMUG00000003357	*SREBF2*	-0.67	2.03E-04	sterol regulatory element binding transcription factor 2
ENSMMUG00000014072	*IER3*	0.68	2.08E-04	immediate early response 3
ENSMMUG00000038454	*H2AFZ*	0.61	2.15E-04	H2A,Z variant histone 1
ENSMMUG00000009247	*OTUD1*	1.16	2.18E-04	OTU deubiquitinase 1
ENSMMUG00000000208	*NRG1*	1.45	2.20E-04	neuregulin 1
ENSMMUG00000012889	*PCYT2*	-0.65	2.34E-04	phosphate cytidylyltransferase 2, ethanolamine
ENSMMUG00000004321	*LAMA3*	-0.90	2.37E-04	laminin subunit alpha 3
ENSMMUG00000013290	*LURAP1L*	1.34	2.37E-04	leucine rich adaptor protein 1 like
ENSMMUG00000016995	*LUM*	-1.74	2.64E-04	lumican
ENSMMUG00000041372	*CD24*	-1.08	2.77E-04	CD24 molecule
ENSMMUG00000006088	*KLF4*	2.71	2.77E-04	KLF transcription factor 4
ENSMMUG00000018653	*KRT19*	-0.95	3.01E-04	keratin 19
ENSMMUG00000017822	*PPL*	-0.70	3.06E-04	periplakin
ENSMMUG00000019514	*UAP1*	1.23	3.06E-04	UDP-N-acetylglucosamine pyrophosphorylase 1
ENSMMUG00000013865	*WARS*	0.92	3.33E-04	tryptophanyl-tRNA synthetase
ENSMMUG00000019849	*BMP2*	1.34	3.37E-04	bone morphogenetic protein 2
ENSMMUG00000047534	*ELOVL6*	-1.12	3.39E-04	ELOVL fatty acid elongase 6
ENSMMUG00000047391	*SRXN1*	1.41	3.47E-04	sulfiredoxin 1
ENSMMUG00000032072	*N/A*	1.28	3.47E-04	N/A
ENSMMUG00000045219	*CEBPB*	1.44	3.54E-04	CCAAT enhancer binding protein beta
ENSMMUG00000013416	*PPP2R2C*	1.16	3.58E-04	protein phosphatase 2 regulatory subunit Bgamma
ENSMMUG00000029123	*URB1*	0.88	3.61E-04	URB1 ribosome biogenesis homolog
ENSMMUG00000010295	*N/A*	1.12	3.61E-04	N/A
ENSMMUG00000021653	*AARS*	0.88	3.75E-04	alanyl-tRNA synthetase 1

ENSMMUG00000007593	*PHKA1*	0.70	3.78E-04	phosphorylase kinase regulatory subunit alpha 1
ENSMMUG00000018014	*MACO1*	0.69	3.81E-04	macoilin 1
ENSMMUG00000003744	*SLC44A2*	-0.66	3.84E-04	solute carrier family 44 member 2
ENSMMUG00000040398	*N/A*	1.34	3.87E-04	N/A
ENSMMUG00000012644	*OSMR*	0.89	4.19E-04	oncostatin M receptor
ENSMMUG00000010662	*SLC16A9*	-1.37	4.19E-04	solute carrier family 16 member 9
ENSMMUG00000001609	*AKAP1*	0.68	4.19E-04	A-kinase anchoring protein 1
ENSMMUG00000021785	*SHMT2*	0.96	4.19E-04	serine hydroxymethyltransferase 2
ENSMMUG00000008911	*ALG2*	0.68	4.19E-04	ALG2 alpha-1,3/1,6-mannosyltransferase
ENSMMUG00000021286	*COL3A1*	-0.73	4.36E-04	collagen type III alpha 1 chain
ENSMMUG00000017215	*TM4SF1*	-0.66	4.61E-04	transmembrane 4 L six family member 1
ENSMMUG00000016848	*SLCO4A1*	1.00	4.81E-04	solute carrier organic anion transporter family member 4A1
ENSMMUG00000008918	*SRPRB*	0.82	4.86E-04	SRP receptor subunit beta
ENSMMUG00000005875	*LRATD2*	-0.77	4.89E-04	LRAT domain containing 2
ENSMMUG00000020707	*LONP1*	0.65	4.89E-04	lon peptidase 1, mitochondria
ENSMMUG00000047109	*HSPA9*	0.67	4.89E-04	heat shock protein family A (Hsp70) member 9
ENSMMUG00000040957	*SPSB1*	1.39	4.89E-04	splA/ryanodine receptor domain and SOCS box containing 1
ENSMMUG00000002052	*N/A*	-0.61	4.89E-04	N/A
ENSMMUG00000009914	*SMAD9*	-0.99	4.91E-04	SMAD family member 9
ENSMMUG00000011038	*FZD2*	-0.68	4.95E-04	frizzled class receptor 2
ENSMMUG00000016387	*ADM*	0.87	4.96E-04	adrenomedullin
ENSMMUG00000020558	*STK38*	-0.71	4.96E-04	serine/threonine kinase 38
ENSMMUG00000004600	*RCAN1*	0.89	5.67E-04	regulator of calcineurin 1
ENSMMUG00000016294	*SYCP2*	1.09	5.67E-04	synaptonemal complex protein 2
ENSMMUG00000003582	*MXRA8*	-0.69	5.92E-04	matrix remodeling associated 8
ENSMMUG00000014818	*MYLK*	-0.78	5.92E-04	myosin light chain kinase
ENSMMUG00000003280	*HBEGF*	0.84	5.95E-04	heparin binding EGF like growth factor
ENSMMUG00000023265	*AKAP12*	1.18	5.96E-04	A-kinase anchoring protein 12
ENSMMUG00000022163	*SLC1A5*	1.51	6.12E-04	solute carrier family 1 member 5
ENSMMUG00000003585	*ALG5*	0.69	6.75E-04	ALG5 dolichyl-phosphate beta-glucosyltransferase
ENSMMUG00000038006	*OTX2*	-0.68	7.37E-04	orthodenticle homeobox 2
ENSMMUG00000022481	*CYP1A1*	2.43	8.03E-04	cytochrome P450 family 1 subfamily A member 1
ENSMMUG00000004935	*PFKP*	0.69	8.40E-04	phosphofructokinase, platelet
ENSMMUG00000032156	*C3AR1*	1.27	8.49E-04	complement C3a receptor 1
ENSMMUG00000039317	*CREB5*	1.75	8.52E-04	cAMP responsive element binding protein 5
ENSMMUG00000020839	*SEPHS2*	0.94	8.52E-04	selenophosphate synthetase 2
ENSMMUG00000019570	*N/A*	-1.12	8.59E-04	N/A
ENSMMUG00000019268	*CD82*	-0.70	8.61E-04	CD82 molecule
ENSMMUG00000008890	*NR1D1*	1.46	8.71E-04	nuclear receptor subfamily 1 group D member 1
ENSMMUG00000002933	*GTPBP2*	1.05	8.71E-04	GTP binding protein 2
ENSMMUG00000007428	*GAS6*	-0.68	8.80E-04	growth arrest specific 6
ENSMMUG00000006156	*TRPM3*	-0.93	9.20E-04	transient receptor potential cation channel subfamily M member 3
ENSMMUG00000021837	*SARS*	0.96	9.21E-04	seryl-tRNA synthetase 1
ENSMMUG00000008134	*DNAJA3*	0.69	9.64E-04	DnaJ heat shock protein family (Hsp40) member A3
ENSMMUG00000016938	*DFNA5*	0.95	9.87E-04	Non-syndromic hearing impairment protein 5 isoform A
ENSMMUG00000019820	*SH3TC2*	-0.82	9.88E-04	SH3 domain and tetratricopeptide repeats 2
ENSMMUG00000001819	*LRRC17*	-0.74	9.93E-04	leucine rich repeat containing 17
ENSMMUG00000012432	*ISG15*	1.10	9.93E-04	ISG15 ubiquitin like modifier
ENSMMUG00000013303	*LRRC4*	-0.98	1.05E-03	leucine rich repeat containing 4
ENSMMUG00000015436	*ALDH1A1*	-0.70	1.05E-03	aldehyde dehydrogenase 1 family member A1

ENSMMUG00000008334	*GPR1*	-0.78	1.05E-03	chemerin chemokine-like receptor 2
ENSMMUG00000017427	*ATP1A3*	0.98	1.10E-03	ATPase Na+/K+ transporting subunit alpha 3
ENSMMUG00000000664	*RRAS2*	0.74	1.14E-03	RAS related 2
ENSMMUG00000013059	*GPR180*	0.88	1.21E-03	G protein-coupled receptor 180
ENSMMUG00000019873	*E2F7*	0.69	1.30E-03	E2F transcription factor 7
ENSMMUG00000022846	*ST6GALNAC4*	0.84	1.30E-03	ST6 N-acetylgalactosaminide alpha-2,6-sialyltransfer-ase 4
ENSMMUG00000015917	*CYP4B1*	-0.86	1.30E-03	cytochrome P450 family 4 subfamily B member 1
ENSMMUG00000012672	*MYO10*	-0.53	1.32E-03	myosin X
ENSMMUG00000001132	*CDKN2B*	-0.74	1.33E-03	cyclin dependent kinase inhibitor 2B
ENSMMUG00000015185	*ERN1*	0.83	1.33E-03	endoplasmic reticulum to nucleus signaling 1
ENSMMUG00000030891	*HYOU1*	0.81	1.40E-03	hypoxia up-regulated 1
ENSMMUG00000003896	*TCF4*	-0.68	1.42E-03	transcription factor 4(
ENSMMUG00000030838	*RAVER2*	0.83	1.42E-03	ribonucleoprotein, PTB binding 2
ENSMMUG00000010203	*ARHGAP6*	-0.62	1.42E-03	Rho GTPase activating protein 6
ENSMMUG00000018565	*PTPN6*	-0.80	1.42E-03	protein tyrosine phosphatase non-receptor type 6
ENSMMUG00000012001	*GTF2A1L*	-0.67	1.42E-03	general transcription factor IIA subunit 1 like
ENSMMUG00000046124	*SLC2A3*	0.86	1.45E-03	solute carrier family 2 (facilitated glucose trans-porter), member 3
ENSMMUG00000011277	*MARS*	0.78	1.45E-03	methionyl-tRNA synthetase 1
ENSMMUG00000012737	*PLXND1*	-0.71	1.56E-03	plexin D1
ENSMMUG00000028737	*FGF21*	2.60	1.64E-03	fibroblast growth factor 21
ENSMMUG00000007831	*HSPD1*	0.60	1.65E-03	heat shock protein family D (Hsp60) member 1
ENSMMUG00000010489	*PTHLH*	2.15	1.65E-03	parathyroid hormone like hormone
ENSMMUG00000010531	*ARHGAP1*	-0.57	1.65E-03	Rho GTPase activating protein 1
ENSMMUG00000021771	*UBA5*	0.63	1.65E-03	ubiquitin like modifier activating enzyme 5
ENSMMUG00000048558	*MXD4*	-0.56	1.88E-03	MAX dimerization protein 4
ENSMMUG00000015817	*TMTC1*	-0.74	1.89E-03	transmembrane O-mannosyltransferase targeting cadherins 1
ENSMMUG00000022921	*SLC2A12*	-0.93	1.89E-03	solute carrier family 2 member 12
ENSMMUG00000012223	*BCAT1*	1.43	1.90E-03	branched chain amino acid transaminase 1
ENSMMUG00000012069	*TGFB3*	-0.80	1.91E-03	transforming growth factor beta 3
ENSMMUG00000019072	*FAM124A*	-0.79	1.92E-03	family with sequence similarity 124 member A
ENSMMUG00000001480	*DAAM2*	-0.55	1.92E-03	dishevelled associated activator of morphogenesis 2
ENSMMUG00000023257	*NFIL3*	1.55	1.96E-03	nuclear factor, interleukin 3 regulated
ENSMMUG00000015370	*PLXDC2*	-0.70	2.02E-03	plexin domain containing 2
ENSMMUG00000048779	*CYBRD1*	-0.57	2.08E-03	Cytochrome b reductase 1
ENSMMUG00000046195	*MIS12*	0.64	2.13E-03	MIS12 kinetochore complex component
ENSMMUG00000016399	*RGL1*	-0.73	2.13E-03	ral guanine nucleotide dissociation stimulator like 1
ENSMMUG00000024570	*LOC114677864*	1.60	2.14E-03	small nucleolar RNA SNORA70
ENSMMUG00000041399	*UBAP1L*	1.38	2.16E-03	ubiquitin associated protein 1 like
ENSMMUG00000007273	*DNM3*	-1.42	2.21E-03	dynamin 3
ENSMMUG00000011719	*SERPINB9*	-0.84	2.21E-03	serpin family B member 9
ENSMMUG00000005670	*RPS6KA2*	0.95	2.22E-03	ribosomal protein S6 kinase A2
ENSMMUG00000048302	*AGFG2*	-0.79	2.23E-03	ArfGAP with FG repeats 2
ENSMMUG00000006855	*TLR4*	0.96	2.31E-03	toll like receptor 4
ENSMMUG00000009112	*ACOT2*	-0.67	2.31E-03	acyl-CoA thioesterase 2
ENSMMUG00000039441	*MAB21L2*	-0.67	2.35E-03	mab-21 like 2
ENSMMUG00000009063	*ETS2*	1.01	2.36E-03	ETS proto-oncogene 2, transcription factor
ENSMMUG00000009097	*MMP11*	-0.77	2.37E-03	matrix metallopeptidase 11
ENSMMUG00000017638	*EMP1*	0.75	2.44E-03	epithelial membrane protein 1
ENSMMUG00000022581	*KCNMB1*	-0.69	2.44E-03	potassium calcium-activated channel subfamily M regulatory beta subunit 1
ENSMMUG00000042787	*N/A*	2.35	2.51E-03	N/A

ENSMMUG00000013797	*GFPT1*	0.91	2.52E-03	glutamine--fructose-6-phosphate transaminase 1
ENSMMUG00000018116	*JAG1*	1.32	2.55E-03	jagged canonical Notch ligand 1
ENSMMUG00000017631	*GPRC5A*	0.87	2.61E-03	G protein-coupled receptor class C group 5 member A
ENSMMUG00000010981	*SLC2A6*	-0.94	2.65E-03	solute carrier family 2 member 6
ENSMMUG00000000265	*ADAMTS9*	1.41	2.67E-03	ADAM metallopeptidase with thrombospondin type 1 motif 9
ENSMMUG00000016666	*CHRDL1*	-0.63	2.67E-03	chordin like 1
ENSMMUG00000045506	*MAFF*	1.47	2.67E-03	MAF bZIP transcription factor F
ENSMMUG00000005994	*ST6GALNAC5*	-0.86	2.70E-03	ST6 N-acetylgalactosaminide alpha-2,6-sialyltransfer-ase 5
ENSMMUG00000014219	*OSGIN1*	1.76	2.70E-03	oxidative stress induced growth inhibitor 1
ENSMMUG00000019083	*TFAP4*	0.74	2.74E-03	transcription factor AP-4
ENSMMUG00000044729	*SLC9B2*	0.65	2.74E-03	solute carrier family 9 member B2
ENSMMUG00000031439	*FBXL2*	-0.63	2.80E-03	F-box and leucine rich repeat protein 2
ENSMMUG00000006187	*ANGPTL7*	-0.66	2.82E-03	angiopoietin like 7
ENSMMUG00000009831	*TBC1D2*	-0.64	2.82E-03	TBC1 domain family member 2
ENSMMUG00000002517	*PTPDC1*	2.44	2.85E-03	protein tyrosine phosphatase domain containing 1
ENSMMUG00000009251	*TSPAN9*	-0.67	2.86E-03	tetraspanin 9
ENSMMUG00000023274	*SULF1*	-0.83	2.88E-03	sulfatase 1
ENSMMUG00000009366	*DOCK2*	-0.54	2.92E-03	dedicator of cytokinesis 2
ENSMMUG00000005136	*TCAF1*	-0.62	2.95E-03	TRPM8 channel associated factor 1
ENSMMUG00000000511	*MSC*	1.14	3.05E-03	musculin
ENSMMUG00000013531	*IPO5*	0.56	3.05E-03	importin 5
ENSMMUG00000000947	*TEX30*	0.70	3.05E-03	testis expressed 30
ENSMMUG00000000345	*CARS*	0.92	3.05E-03	cysteinyl-tRNA synthetase 1
ENSMMUG00000022066	*UTP25*	0.63	3.05E-03	UTP25 small subunit processor component
ENSMMUG00000019126	*PNP*	0.77	3.08E-03	purine nucleoside phosphorylase
ENSMMUG00000007284	*SUN2*	-0.57	3.09E-03	Sad1 and UNC84 domain containing 2
ENSMMUG00000011328	*CPEB1*	0.65	3.10E-03	cytoplasmic polyadenylation element binding pro-tein 1
ENSMMUG00000003301	*TEF*	-0.75	3.22E-03	TEF transcription factor, PAR bZIP family member
ENSMMUG00000020954	*CDC25A*	0.69	3.22E-03	cell division cycle 25A
ENSMMUG00000005221	*ADAMTS15*	0.89	3.22E-03	ADAM metallopeptidase with thrombospondin type 1 motif 15
ENSMMUG00000023810	*MARS2*	0.97	3.22E-03	methionyl-tRNA synthetase 2, mitochondrial
ENSMMUG00000004475	*MMP2*	-0.59	3.22E-03	matrix metallopeptidase 2
ENSMMUG00000023042	*SPCS2*	0.61	3.35E-03	signal peptidase complex subunit 2
ENSMMUG00000010685	*OLFML2B*	-0.92	3.35E-03	olfactomedin like 2B
ENSMMUG00000039960	*LOC106998872*	1.82	3.42E-03	N/A
ENSMMUG00000013048	*NPR3*	-1.39	3.58E-03	natriuretic peptide receptor 3
ENSMMUG00000013366	*JUP*	-0.80	3.58E-03	junction plakoglobin
ENSMMUG00000007906	*CDC42EP3*	0.60	3.63E-03	CDC42 effector protein 3
ENSMMUG00000001583	*FSCN2*	-0.94	3.63E-03	fascin actin-bundling protein 2, retinal
ENSMMUG00000022963	*SLC25A38*	0.71	3.65E-03	solute carrier family 25 member 38
ENSMMUG00000001850	*SPARC*	-0.60	3.81E-03	secreted protein acidic and cysteine rich
ENSMMUG00000005915	*DYNC1I1*	-0.71	3.83E-03	dynein cytoplasmic 1 intermediate chain 1
ENSMMUG00000011798	*LOC705696*	0.58	3.96E-03	eukaryotic translation initiation factor 3 subunit C
ENSMMUG00000021590	*PCK2*	0.94	3.96E-03	phosphoenolpyruvate carboxykinase 2, mitochon-drial
ENSMMUG00000021074	*RASGEF1B*	1.55	3.97E-03	RasGEF domain family member 1B
ENSMMUG00000048864	*ROGDI*	-0.64	4.06E-03	rogdi atypical leucine zipper
ENSMMUG00000017946	*RND1*	2.49	4.06E-03	Rho family GTPase 1
ENSMMUG00000021296	*EML6*	0.79	4.08E-03	EMAP like 6

ENSMMUG00000014175	*SPRY4*	1.38	4.10E-03	sprouty RTK signaling antagonist 4
ENSMMUG00000023393	*LYAR*	0.65	4.24E-03	Ly1 antibody reactive
ENSMMUG00000015799	*TLL1*	-0.66	4.27E-03	tolloid like 1
ENSMMUG00000046090	*ZNF488*	-0.74	4.33E-03	zinc finger protein 488
ENSMMUG00000011907	*RF02251*	-0.86	4.33E-03	N/A
ENSMMUG00000011294	*LSR*	0.62	4.34E-03	lipolysis stimulated lipoprotein receptor
ENSMMUG00000048483	*RF00015*	1.59	4.50E-03	RNA, U4 small nuclear 2
ENSMMUG00000009901	*LDLR*	-0.80	4.65E-03	low density lipoprotein receptor
ENSMMUG00000003237	*ID2*	-0.70	4.65E-03	inhibitor of DNA binding 2
ENSMMUG00000003611	*HMOX2*	0.78	4.65E-03	heme oxygenase 2
ENSMMUG00000002295	*CRABP2*	1.45	4.69E-03	cellular retinoic acid binding protein 2
ENSMMUG00000002182	*PISD*	0.61	4.73E-03	phosphatidylserine decarboxylase
ENSMMUG00000003511	*CLIC5*	-1.08	4.79E-03	chloride intracellular channel 5
ENSMMUG00000008924	*PLLP*	-0.87	4.93E-03	plasmolipin
ENSMMUG00000012427	*FBXL13*	-0.64	5.04E-03	F-box and leucine rich repeat protein 13
ENSMMUG00000014757	*SYT17*	-0.67	5.10E-03	synaptotagmin 17
ENSMMUG00000004179	*LIMS2*	-1.20	5.11E-03	LIM zinc finger domain containing 2
ENSMMUG00000015785	*AMPD3*	0.78	5.17E-03	adenosine monophosphate deaminase 3
ENSMMUG00000039576	*N/A*	0.97	5.35E-03	N/A
ENSMMUG00000014784	*DSP*	-0.57	5.38E-03	desmoplakin
ENSMMUG00000045851	*N/A*	0.93	5.42E-03	N/A
ENSMMUG00000007231	*ZSCAN31*	-0.76	5.49E-03	zinc finger and SCAN domain containing 31
ENSMMUG00000011999	*SLC38A1*	0.83	5.49E-03	solute carrier family 38 member 1
ENSMMUG00000001952	*SRGAP1*	1.40	5.88E-03	SLIT-ROBO Rho GTPase activating protein 1
ENSMMUG00000018791	*MOCOS*	0.78	5.93E-03	molybdenum cofactor sulfurase
ENSMMUG00000012068	*SIPA1L2*	2.01	6.09E-03	signal induced proliferation associated 1 like 2
ENSMMUG00000020987	*FABP3*	-0.52	6.22E-03	fatty acid binding protein 4
ENSMMUG00000005093	*KLHL32*	-0.81	6.32E-03	kelch like family member 32
ENSMMUG00000018880	*ABHD17B*	-0.83	6.69E-03	abhydrolase domain containing 17B, depalmitoylase
ENSMMUG00000001183	*NOLC1*	0.50	6.73E-03	nucleolar and coiled-body phosphoprotein 1
ENSMMUG00000013808	*SH3BP4*	0.62	6.82E-03	SH3 domain binding protein 4
ENSMMUG00000012634	*GTF2F2*	0.57	6.92E-03	general transcription factor IIF subunit 2
ENSMMUG00000006057	*NTMT1*	0.55	6.95E-03	N-terminal Xaa-Pro-Lys N-methyltransferase 1
ENSMMUG00000008003	*LRRC8C*	0.80	6.99E-03	leucine rich repeat containing 8 VRAC subunit C
ENSMMUG00000008653	*FOSL1*	0.74	7.04E-03	FOS like 1, AP-1 transcription factor subunit
ENSMMUG00000000145	*PDPN*	-0.58	7.13E-03	podoplanin
ENSMMUG00000005722	*PDCD4*	-0.78	7.27E-03	programmed cell death 4
ENSMMUG00000001996	*F11R*	-0.74	7.27E-03	F11 receptor
ENSMMUG00000017204	*ADGRE5*	-0.53	7.27E-03	adhesion G protein-coupled receptor E5
ENSMMUG00000004496	*MIEF1*	0.55	7.30E-03	mitochondrial elongation factor 1
ENSMMUG00000016040	*RND3*	0.70	7.34E-03	Rho family GTPase 3
ENSMMUG00000010114	*TMEM63A*	-0.51	7.37E-03	transmembrane protein 63A
ENSMMUG00000005785	*RIN2*	-0.61	7.37E-03	Ras and Rab interactor 2
ENSMMUG00000045396	*SELENBP1*	-0.60	7.63E-03	selenium binding protein 1
ENSMMUG00000021247	*TGM2*	-0.77	7.70E-03	transglutaminase 2
ENSMMUG00000018925	*ALS2CL*	-0.60	7.70E-03	ALS2 C-terminal like
ENSMMUG00000013640	*ZBTB43*	0.68	7.72E-03	zinc finger and BTB domain containing 43
ENSMMUG00000007401	*ARRDC2*	-0.59	7.73E-03	arrestin domain containing 2
ENSMMUG00000022019	*CTSO*	-0.49	7.74E-03	cathepsin O
ENSMMUG00000014372	*BMF*	-0.74	7.92E-03	Bcl2 modifying factor
ENSMMUG00000007729	*ILVBL*	-0.52	7.92E-03	ilvB acetolactate synthase like
ENSMMUG00000023491	*LGR4*	-0.70	8.09E-03	leucine rich repeat containing G protein-coupled receptor 4
ENSMMUG00000008100	*SLC6A3*	-0.58	8.35E-03	solute carrier family 6 member 3

ENSMMUG00000005198	*IRF1*	0.67	8.35E-03	interferon regulatory factor 1
ENSMMUG00000010808	*PREB*	0.63	8.42E-03	prolactin regulatory element binding
ENSMMUG00000002390	*C1H1orf43*	0.53	8.42E-03	chromosome 1 C1orf43 homolog
ENSMMUG00000017300	*PFDN4*	0.54	8.43E-03	prefoldin subunit 4
ENSMMUG00000002640	*TRPC4*	-0.86	8.48E-03	transient receptor potential cation channel subfamily C member 4
ENSMMUG00000003974	*EBNA1BP2*	0.50	8.48E-03	EBNA1 binding protein 2
ENSMMUG00000013997	*GREB1L*	-0.68	8.48E-03	GREB1 like retinoic acid receptor coactivator
ENSMMUG00000019270	*LOC712661*	0.84	8.48E-03	perilipin-2
ENSMMUG00000022873	*MTO1*	0.54	8.48E-03	mitochondrial tRNA translation optimization 1
ENSMMUG00000030786	*SERPINB8*	0.94	8.48E-03	serpin family B member 8
ENSMMUG00000015607	*SLC39A13*	-0.46	8.52E-03	solute carrier family 39 member 13
ENSMMUG00000011557	*PDGFRB*	-0.66	8.55E-03	platelet derived growth factor receptor beta
ENSMMUG00000021484	*NPC1*	-0.49	8.63E-03	NPC intracellular cholesterol transporter 1
ENSMMUG00000001847	*GBP6*	1.38	8.71E-03	guanylate binding protein family member 6
ENSMMUG00000020157	*CNTN4*	2.55	8.71E-03	contactin 4
ENSMMUG00000042891	*QPRT*	-0.87	8.71E-03	quinolinate phosphoribosyltransferase
ENSMMUG00000023621	*DDX27*	0.54	9.13E-03	DEAD-box helicase 27
ENSMMUG00000003587	*KLRC1*	1.97	9.20E-03	killer cell lectin like receptor C1
ENSMMUG00000024424	*LOC114679145*	2.38	9.30E-03	small nucleolar RNA SNORD63
ENSMMUG00000044591	*N/A*	-0.72	9.62E-03	N/A
ENSMMUG00000003708	*PDE7B*	0.93	9.92E-03	phosphodiesterase 7B
ENSMMUG00000004383	*ADGRF3*	-0.64	9.95E-03	adhesion G protein-coupled receptor F3
ENSMMUG00000022750	*ID1*	-0.77	9.97E-03	inhibitor of DNA binding 1
ENSMMUG00000004403	*ZBTB21*	0.75	9.97E-03	zinc finger and BTB domain containing 21
ENSMMUG00000018053	*DPP7*	-0.45	9.97E-03	dipeptidyl peptidase 7
ENSMMUG00000008633	*ANK2*	-0.56	1.01E-02	ankyrin 2
ENSMMUG00000015968	*FKBP4*	0.57	1.01E-02	FKBP prolyl isomerase 4
ENSMMUG00000007405	*CMPK2*	1.17	1.01E-02	cytidine/uridine monophosphate kinase 2
ENSMMUG00000048768	*N/A*	-0.64	1.01E-02	N/A
ENSMMUG00000004751	*PLA2G15*	-0.67	1.01E-02	phospholipase A2 group XV
ENSMMUG00000014685	*PDK2*	-0.53	1.01E-02	pyruvate dehydrogenase kinase 2
ENSMMUG00000010147	*ALDH4A1*	-0.65	1.01E-02	aldehyde dehydrogenase 4 family member A1
ENSMMUG00000003316	*MAN2A1*	0.62	1.05E-02	mannosidase alpha class 2A member 1
ENSMMUG00000041440	*N/A*	1.25	1.05E-02	N/A
ENSMMUG00000012569	*TMEM132D*	0.65	1.05E-02	transmembrane protein 132D
ENSMMUG00000039045	*NGF*	0.60	1.05E-02	nerve growth factor
ENSMMUG00000013342	*HPSE*	0.68	1.05E-02	heparanase
ENSMMUG00000004196	*MGARP*	-1.06	1.07E-02	mitochondria localized glutamic acid rich protein
ENSMMUG00000019661	*TPST2*	-0.51	1.08E-02	tyrosylprotein sulfotransferase 2
ENSMMUG00000023450	*PIK3IP1*	-0.84	1.08E-02	phosphoinositide-3-kinase interacting protein 1
ENSMMUG00000017017	*FYN*	0.65	1.08E-02	FYN proto-oncogene, Src family tyrosine kinase
ENSMMUG00000008793	*SPP1*	-0.67	1.08E-02	secreted phosphoprotein 1
ENSMMUG00000014230	*FMO4*	-0.55	1.10E-02	flavin containing dimethylaniline monoxygenase 4
ENSMMUG00000012138	*RORC*	-0.49	1.11E-02	RAR related orphan receptor C
ENSMMUG00000001127	*TIMP4*	1.43	1.11E-02	TIMP metallopeptidase inhibitor 4
ENSMMUG00000023533	*SERINC5*	-0.51	1.12E-02	serine incorporator 5
ENSMMUG00000010814	*TRIB3*	1.55	1.13E-02	tribbles pseudokinase 3
ENSMMUG00000012600	*HAX1*	0.62	1.13E-02	HCLS1 associated protein X-1
ENSMMUG00000005788	*LOC693799*	1.15	1.14E-02	mitochondrial import inner membrane translocase subunit Tim9
ENSMMUG00000023365	*ME1*	-0.53	1.15E-02	malic enzyme 1
ENSMMUG00000012592	*UROS*	-0.51	1.15E-02	uroporphyrinogen III synthase
ENSMMUG00000021407	*LOC706619*	-0.51	1.15E-02	transducin-like enhancer protein 4

ENSMMUG00000008539	*TRAF4*	-0.50	1.15E-02	TNF receptor associated factor 4
ENSMMUG00000008033	*IFRD1*	0.98	1.21E-02	interferon related developmental regulator 1
ENSMMUG00000002357	*TMEM2*	-0.53	1.21E-02	cell migration inducing hyaluronidase 2
ENSMMUG00000022403	*LRRC32*	-0.84	1.21E-02	leucine rich repeat containing 32
ENSMMUG00000010279	*SLC1A4*	0.65	1.21E-02	solute carrier family 1 member 4
ENSMMUG00000046776	*N/A*	-1.19	1.21E-02	N/A
ENSMMUG00000047621	*TM4SF18*	-0.99	1.23E-02	transmembrane 4 L six family member 18
ENSMMUG00000021225	*SAT1*	0.96	1.24E-02	spermidine/spermine N1-acetyltransferase 1
ENSMMUG00000010813	*GAR1*	0.85	1.24E-02	GAR1 ribonucleoprotein
ENSMMUG00000015329	*GTPBP4*	0.55	1.24E-02	GTP binding protein 4
ENSMMUG00000008335	*MX1*	1.73	1.24E-02	MX dynamin like GTPase 1
ENSMMUG00000029011	*UBXN8*	0.56	1.24E-02	UBX domain protein 8
ENSMMUG00000040972	*KCNK3*	-1.01	1.24E-02	potassium two pore domain channel subfamily K member 3
ENSMMUG00000044029	*C1QTNF6*	-0.76	1.25E-02	C1q and TNF related 6
ENSMMUG00000016418	*CBX4*	0.62	1.25E-02	chromobox 4
ENSMMUG00000021764	*MMP10*	1.54	1.27E-02	matrix metallopeptidase 10
ENSMMUG00000012895	*PYCR1*	0.82	1.28E-02	pyrroline-5-carboxylate reductase 1
ENSMMUG00000005431	*SLC25A1*	-0.48	1.29E-02	solute carrier family 25 member 1
ENSMMUG00000007052	*MMP15*	0.87	1.31E-02	matrix metallopeptidase 15
ENSMMUG00000011161	*STXBP5L*	-0.61	1.35E-02	syntaxin binding protein 5L
ENSMMUG00000041731	*TMEM86A*	-0.46	1.37E-02	transmembrane protein 86A
ENSMMUG00000002038	*ST3GAL1*	0.74	1.38E-02	ST3 beta-galactoside alpha-2,3-sialyltransferase 1
ENSMMUG00000012531	*CEMIP*	-0.75	1.38E-02	cell migration inducing hyaluronidase 1
ENSMMUG00000014498	*CACNA1E*	1.06	1.38E-02	calcium voltage-gated channel subunit alpha1 E
ENSMMUG00000015158	*SLC6A9*	1.46	1.39E-02	solute carrier family 6 member 9
ENSMMUG00000000828	*GAB1*	-0.60	1.45E-02	GRB2 associated binding protein 1
ENSMMUG00000010594	*PTGFR*	1.36	1.45E-02	prostaglandin F receptor
ENSMMUG00000003189	*PPIF*	0.75	1.46E-02	peptidylprolyl isomerase F
ENSMMUG00000044497	*N/A*	1.07	1.46E-02	N/A
ENSMMUG00000019850	*SLC47A1*	0.61	1.48E-02	solute carrier family 47 member 1
ENSMMUG00000002700	*ALOXE3*	1.79	1.48E-02	arachidonate lipoxygenase 3
ENSMMUG00000003289	*ITGB3*	1.16	1.48E-02	integrin subunit beta 3
ENSMMUG00000019859	*PRKG2*	-1.22	1.48E-02	protein kinase cGMP-dependent 2
ENSMMUG00000020408	*HPGD*	-0.89	1.49E-02	15-hydroxyprostaglandin dehydrogenase
ENSMMUG00000014618	*SEC23B*	0.51	1.49E-02	SEC23 homolog B, COPII coat complex component
ENSMMUG00000012878	*SPATA9*	2.43	1.49E-02	spermatogenesis associated 9
ENSMMUG00000001213	*NDRG4*	0.49	1.49E-02	NDRG family member 4
ENSMMUG00000004872	*TMEM220*	-0.72	1.49E-02	transmembrane protein 220
ENSMMUG00000008869	*TNFAIP3*	0.89	1.49E-02	TNF alpha induced protein 3
ENSMMUG00000004594	*CGRRF1*	0.56	1.51E-02	cell growth regulator with ring finger domain 1
ENSMMUG00000020941	*LOC693438*	0.89	1.52E-02	heat shock 60kDa protein 1 (chaperonin)-like
ENSMMUG00000022927	*RCAN3*	-0.67	1.53E-02	RCAN family member 3
ENSMMUG00000002678	*ITGB6*	-1.17	1.55E-02	integrin subunit beta 6
ENSMMUG00000010393	*RAC1*	-0.58	1.55E-02	Rac family small GTPase 1
ENSMMUG00000010895	*CDH3*	-0.68	1.55E-02	cadherin 3
ENSMMUG00000047800	*PFKFB3*	0.64	1.55E-02	6-phosphofructo-2-kinase/fructose-2,6-biphosphatase 3
ENSMMUG00000011115	*TYR*	-0.60	1.55E-02	tyrosinase
ENSMMUG00000041795	*CROCC2*	-1.11	1.55E-02	ciliary rootlet coiled-coil, rootletin family member 2
ENSMMUG00000001005	*DDX21*	0.51	1.55E-02	DExD-box helicase 21
ENSMMUG00000009429	*FAM102B*	-0.74	1.56E-02	EEIG family member 2
ENSMMUG00000000529	*GPC6*	-0.67	1.61E-02	glypican 6
ENSMMUG00000016030	*TRIM25*	0.62	1.61E-02	tripartite motif containing 25

ENSMMUG00000005790	*KLHL29*	0.55	1.61E-02	kelch like family member 29
ENSMMUG00000009238	*IL1RL1*	2.29	1.66E-02	interleukin 1 receptor like 1
ENSMMUG00000001694	*FZD8*	-0.59	1.67E-02	frizzled class receptor 8
ENSMMUG00000007798	*CLMP*	1.05	1.68E-02	CXADR like membrane protein
ENSMMUG00000009528	*THSD1*	0.89	1.71E-02	thrombospondin type 1 domain containing
ENSMMUG00000006284	*MBOAT2*	-0.54	1.71E-02	membrane bound O-acyltransferase domain containing 2
ENSMMUG00000021037	*AK2*	0.54	1.71E-02	adenylate kinase 2
ENSMMUG00000002103	*DNER*	1.67	1.73E-02	delta/notch like EGF repeat containing
ENSMMUG00000003839	*MAPKBP1*	-0.48	1.74E-02	mitogen-activated protein kinase binding protein 1
ENSMMUG00000020086	*POR*	0.57	1.75E-02	cytochrome p450 oxidoreductase
ENSMMUG00000031911	*KRT18*	0.52	1.76E-02	keratin 18
ENSMMUG00000000343	*SCN3B*	2.30	1.76E-02	sodium voltage-gated channel beta subunit 3
ENSMMUG00000012100	*MYOM1*	-0.68	1.77E-02	myomesin 1
ENSMMUG00000019892	*CDH15*	-1.06	1.77E-02	cadherin 15
ENSMMUG00000040286	*NAPEPLD*	-0.55	1.77E-02	N-acyl phosphatidylethanolamine phospholipase D
ENSMMUG00000004236	*N/A*	-0.89	1.77E-02	N/A
ENSMMUG00000042378	*PEAK1*	-0.53	1.79E-02	pseudopodium enriched atypical kinase 1
ENSMMUG00000018572	*GHITM*	0.53	1.82E-02	growth hormone inducible transmembrane protein
ENSMMUG00000040044	*SEMA3A*	-0.90	1.82E-02	semaphorin 3A
ENSMMUG00000007555	*RF00056*	1.43	1.82E-02	N/A
ENSMMUG00000020374	*NFKBIA*	0.55	1.83E-02	NFKB inhibitor alpha
ENSMMUG00000016840	*SOWAHC*	0.90	1.83E-02	sosondowah ankyrin repeat domain family member C
ENSMMUG00000017179	*MAP2K1*	0.46	1.83E-02	mitogen-activated protein kinase kinase 1
ENSMMUG00000000980	*YPEL3*	-0.50	1.83E-02	yippee like 3
ENSMMUG00000005220	*SYNM*	-0.58	1.87E-02	synemin
ENSMMUG00000017588	*NRP1*	-0.51	1.87E-02	neuropilin 1
ENSMMUG00000043224	*PRRX1*	-0.52	1.89E-02	paired related homeobox 1
ENSMMUG00000017101	*CTPS1*	0.49	1.91E-02	CTP synthase 1
ENSMMUG00000014992	*RAB2B*	0.45	1.91E-02	RAB2B, member RAS oncogene family
ENSMMUG00000020365	*ARHGAP18*	-0.62	1.91E-02	Rho GTPase activating protein 18
ENSMMUG00000043719	*C1H1orf216*	-0.60	1.91E-02	chromosome 1 open reading frame
ENSMMUG00000011902	*OSTC*	0.55	1.92E-02	oligosaccharyltransferase complex non-catalytic subunit
ENSMMUG00000007814	*GPM6A*	-0.54	1.92E-02	glycoprotein M6A
ENSMMUG00000012848	*XPR1*	-0.51	1.93E-02	xenotropic and polytropic retrovirus receptor 1
ENSMMUG00000015676	*CDSN*	-1.36	1.94E-02	corneodesmosin
ENSMMUG00000024764	*LOC114680488*	0.81	1.96E-02	small nucleolar RNA SNORA72
ENSMMUG00000018411	*INSL4*	-0.94	1.97E-02	insulin like 4
ENSMMUG00000002614	*SLIT2*	1.13	1.97E-02	slit guidance ligand 2
ENSMMUG00000003721	*KLF15*	1.03	1.97E-02	KLF transcription factor 15
ENSMMUG00000015303	*ABCA7*	-0.51	1.98E-02	ATP binding cassette subfamily A member 7
ENSMMUG00000045968	*N/A*	0.71	1.98E-02	N/A
ENSMMUG00000044239	*H3F3A*	-0.59	1.99E-02	histone H3,3
ENSMMUG00000003569	*ARRDC3*	-0.53	1.99E-02	arrestin domain containing 3
ENSMMUG00000048596	*HILPDA*	0.72	1.99E-02	hypoxia inducible lipid droplet associated
ENSMMUG00000014514	*CXCL2*	1.30	1.99E-02	C-X-C motif chemokine ligand 2
ENSMMUG00000000198	*COLGALT1*	-0.56	1.99E-02	collagen beta(1-O)galactosyltransferase 1
ENSMMUG00000020148	*BAG1*	0.59	2.01E-02	BAG cochaperone 1
ENSMMUG00000003709	*MTFR2*	0.53	2.01E-02	mitochondrial fission regulator 2
ENSMMUG00000020324	*SMIM14*	0.51	2.02E-02	small integral membrane protein 14
ENSMMUG00000022301	*STK38L*	-0.69	2.05E-02	serine/threonine kinase 38 like
ENSMMUG00000010028	*RAG2*	-1.80	2.05E-02	recombination activating 2

ENSMMUG00000018268	*LZTS3*	0.57	2.05E-02	leucine zipper tumor suppressor family member 3
ENSMMUG00000023248	*AHNAK*	-0.48	2.05E-02	AHNAK nucleoprotein
ENSMMUG00000017252	*SPATA20*	-0.44	2.09E-02	spermatogenesis associated 20
ENSMMUG00000021702	*CREB3L1*	-0.60	2.09E-02	cAMP responsive element binding protein 3 like 1
ENSMMUG00000018571	*COA6*	0.86	2.09E-02	cytochrome c oxidase assembly factor 6
ENSMMUG00000016897	*SECTM1*	2.02	2.11E-02	secreted and transmembrane 1
ENSMMUG00000019774	*MTHFD1L*	0.54	2.14E-02	methylenetetrahydrofolate dehydrogenase (NADP+ dependent) 1 like
ENSMMUG00000022693	*SLC8A1*	-0.45	2.16E-02	solute carrier family 8 member A1
ENSMMUG00000015508	*OXNAD1*	0.48	2.16E-02	oxidoreductase NAD binding domain containing 1
ENSMMUG00000016872	*EFEMP1*	-0.44	2.19E-02	EGF containing fibulin extracellular matrix protein 1
ENSMMUG00000004409	*ZNF608*	-0.60	2.20E-02	zinc finger protein 608
ENSMMUG00000002147	*SSR1*	0.47	2.21E-02	signal sequence receptor subunit 1
ENSMMUG00000047327	*IGFBP7*	-0.51	2.21E-02	insulin like growth factor binding protein 7
ENSMMUG00000014011	*SLC38A11*	-0.51	2.21E-02	solute carrier family 38 member 11
ENSMMUG00000048391	*DDRGK1*	0.50	2.21E-02	DDRGK domain containing 1
ENSMMUG00000005009	*LOC106996872*	0.51	2.21E-02	translocon-associated protein subunit gamma pseudogene
ENSMMUG00000019018	*SMAD6*	-1.10	2.23E-02	SMAD family member 6
ENSMMUG00000024487	*LOC114672518*	0.64	2.24E-02	small nucleolar RNA SNORD22
ENSMMUG00000013494	*EZH2*	0.54	2.32E-02	enhancer of zeste 2 polycomb repressive complex 2 subunit
ENSMMUG00000016970	*SSR3*	0.49	2.32E-02	signal sequence receptor subunit 3
ENSMMUG00000028673	*TUBA1A*	-0.42	2.35E-02	tubulin alpha-1C chain
ENSMMUG00000009788	*ND5*	-0.53	2.35E-02	NADH ubiquinone oxidoreductase core subunit 5
ENSMMUG00000041498	*TRAF1*	0.82	2.35E-02	TNF receptor associated factor 1
ENSMMUG00000048076	*TNFRSF10B*	0.60	2.36E-02	TNF receptor superfamily member 10b
ENSMMUG00000016009	*LOC723733*	0.55	2.38E-02	eukaryotic translation elongation factor 1 gamma-like
ENSMMUG00000015093	*VLDLR*	0.51	2.38E-02	very low density lipoprotein receptor
ENSMMUG00000015585	*GPT*	-1.14	2.42E-02	glutamic--pyruvic transaminase
ENSMMUG00000003909	*ST7*	0.49	2.45E-02	suppression of tumorigenicity 7
ENSMMUG00000021722	*FTL*	0.60	2.45E-02	ferritin light chain
ENSMMUG00000048823	*RHBDF2*	0.73	2.46E-02	rhomboid 5 homolog 2
ENSMMUG00000018973	*N/A*	-0.53	2.46E-02	N/A
ENSMMUG00000014414	*ABHD2*	-0.49	2.47E-02	abhydrolase domain containing 2, acylglycerol lipase
ENSMMUG00000030744	*ARID5A*	0.96	2.49E-02	AT-rich interaction domain 5A
ENSMMUG00000017123	*PLA2G12A*	0.56	2.50E-02	phospholipase A2 group XIIA
ENSMMUG00000014430	*FICD*	1.04	2.50E-02	FIC domain protein adenylyltransferase
ENSMMUG00000008177	*FOSB*	2.81	2.50E-02	FosB proto-oncogene, AP-1 transcription factor subunit
ENSMMUG00000018639	*MEX3B*	-0.98	2.50E-02	mex-3 RNA binding family member B
ENSMMUG00000042580	*ARF1*	0.47	2.50E-02	ADP ribosylation factor 1
ENSMMUG00000016053	*ZNF226*	-0.56	2.50E-02	zinc finger protein 226
ENSMMUG00000047704	*LIX1L*	-0.51	2.50E-02	limb and CNS expressed 1 like
ENSMMUG00000017915	*PAEP*	-1.17	2.50E-02	progestagen-associated endometrial protein
ENSMMUG00000046424	*C11H12orf75*	-0.47	2.54E-02	chromosome 11 C12orf75 homolog
ENSMMUG00000007665	*HCFC1R1*	2.18	2.54E-02	host cell factor C1 regulator 1
ENSMMUG00000022672	*HTRA3*	-1.38	2.54E-02	HtrA serine peptidase 3(HTRA3)
ENSMMUG00000012618	*SERPING1*	-0.48	2.55E-02	serpin family G member 1
ENSMMUG00000022394	*EGFR*	0.62	2.55E-02	epidermal growth factor receptor
ENSMMUG00000042327	*N/A*	1.44	2.56E-02	N/A
ENSMMUG00000020208	*ELOVL4*	0.99	2.57E-02	ELOVL fatty acid elongase 4
ENSMMUG00000007400	*TP53I3*	0.65	2.57E-02	tumor protein p53 inducible protein 3

ENSMMUG00000001283	*ZFP36L1*	-0.42	2.57E-02	ZFP36 ring finger protein like 1
ENSMMUG00000044603	*SIAH1*	0.57	2.57E-02	siah E3 ubiquitin protein ligase 1
ENSMMUG00000019994	*SDCCAG8*	-0.46	2.57E-02	SHH signaling and ciliogenesis regulator SDCCAG8
ENSMMUG00000008094	*DUSP4*	0.98	2.57E-02	dual specificity phosphatase 4
ENSMMUG00000014952	*NUP210L*	0.79	2.58E-02	nucleoporin 210 like
ENSMMUG00000007918	*ZNHIT3*	0.66	2.58E-02	zinc finger HIT-type containing 3
ENSMMUG00000022124	*STON2*	-0.57	2.64E-02	stonin 2
ENSMMUG00000001031	*TMEM231*	0.52	2.64E-02	transmembrane protein 231
ENSMMUG00000016074	*MYCL*	1.10	2.64E-02	MYCL proto-oncogene, bHLH transcription factor
ENSMMUG00000016223	*ABHD5*	0.66	2.64E-02	abhydrolase domain containing 5, lysophosphatidic acid acyltransferase
ENSMMUG00000001564	*LFNG*	-0.94	2.64E-02	LFNG O-fucosylpeptide 3-beta-N-acetylglucosami-nyltransferase
ENSMMUG00000018379	*FAM13A*	-0.56	2.65E-02	family with sequence similarity 13 member A
ENSMMUG00000010141	*DEPDC1B*	0.83	2.65E-02	DEP domain containing 1B
ENSMMUG00000047033	*ULBP3*	1.04	2.67E-02	UL16-binding protein 3
ENSMMUG00000008247	*MXRA5*	-0.49	2.67E-02	matrix remodeling associated 5
ENSMMUG00000009615	*IMP4*	0.51	2.67E-02	IMP U3 small nucleolar ribonucleoprotein 4
ENSMMUG00000001172	*SOHLH2*	1.44	2.68E-02	spermatogenesis and oogenesis specific basic helix-loop-helix 2
ENSMMUG00000005778	*TXNRD1*	0.53	2.70E-02	thioredoxin reductase 1
ENSMMUG00000012091	*FOXC1*	1.01	2.70E-02	forkhead box C1
ENSMMUG00000019106	*MCAM*	0.63	2.74E-02	melanoma cell adhesion molecule
ENSMMUG00000018601	*DDR1*	-0.49	2.74E-02	discoidin domain receptor tyrosine kinase 1
ENSMMUG00000004764	*CDC14A*	0.84	2.74E-02	cell division cycle 14A
ENSMMUG00000047349	*UPK3B*	-0.51	2.74E-02	uroplakin 3B
ENSMMUG00000042176	*ERRFI1*	0.86	2.75E-02	ERBB receptor feedback inhibitor 1
ENSMMUG00000042036	*GNG3*	-2.24	2.75E-02	G protein subunit gamma 3
ENSMMUG00000017745	*ANGPT1*	-1.01	2.77E-02	angiopoietin 1
ENSMMUG00000021645	*RORA*	0.90	2.77E-02	RAR related orphan receptor A
ENSMMUG00000044139	*TAX1BP3*	-0.40	2.77E-02	Tax1 binding protein 3
ENSMMUG00000011757	*CHST10*	0.63	2.77E-02	carbohydrate sulfotransferase 10
ENSMMUG00000018192	*ERO1B*	0.61	2.77E-02	endoplasmic reticulum oxidoreductase 1 beta
ENSMMUG00000023390	*WWC3*	-0.42	2.77E-02	WWC family member 3
ENSMMUG00000016290	*NUP188*	0.45	2.77E-02	nucleoporin 188
ENSMMUG00000010405	*FAM171B*	0.69	2.81E-02	family with sequence similarity 171 member B
ENSMMUG00000011262	*PDE7A*	-0.61	2.82E-02	phosphodiesterase 7A
ENSMMUG00000031713	*TMEM19*	-0.58	2.90E-02	transmembrane protein 19
ENSMMUG00000020948	*LOC106995399*	0.65	2.90E-02	cytochrome c-like
ENSMMUG00000012324	*LOC713150*	0.60	2.90E-02	aldo-keto reductase family 1 member B10-like
ENSMMUG00000022386	*NXT1*	0.62	2.96E-02	nuclear transport factor 2 like export factor 1
ENSMMUG00000006582	*PLK3*	1.08	2.97E-02	polo like kinase 3
ENSMMUG00000012511	*GNL3*	0.42	2.99E-02	G protein nucleolar 3
ENSMMUG00000018485	*METTL7A*	-0.48	3.01E-02	methyltransferase like 7A
ENSMMUG00000013020	*GSN*	-0.41	3.06E-02	gelsolin
ENSMMUG00000009012	*FAM3A*	-0.43	3.07E-02	FAM3 metabolism regulating signaling molecule A
ENSMMUG00000018901	*LRRC59*	0.54	3.07E-02	leucine rich repeat containing 59
ENSMMUG00000017234	*FGD6*	-0.51	3.07E-02	FYVE, RhoGEF and PH domain containing 6
ENSMMUG00000010653	*MFSD12*	-0.48	3.07E-02	major facilitator superfamily domain containing 12
ENSMMUG00000010514	*SLC6A20*	-0.70	3.07E-02	solute carrier family 6 member 20
ENSMMUG00000017856	*APH1B*	-0.50	3.10E-02	Aph-1 homolog B, gamma-secretase subunit
ENSMMUG00000018683	*TNS2*	-0.49	3.10E-02	tensin 2
ENSMMUG00000000318	*NOP2*	0.55	3.10E-02	NOP2 nucleolar protein
ENSMMUG00000001151	*MTUS1*	-0.57	3.10E-02	microtubule associated scaffold protein 1

ENSMMUG00000016552	*INF2*	-0.55	3.11E-02	inverted formin 2
ENSMMUG00000011497	*WDR44*	-0.47	3.12E-02	WD repeat domain 44
ENSMMUG00000006657	*MYPN*	0.81	3.12E-02	myopalladin
ENSMMUG00000006867	*FEN1*	0.50	3.12E-02	flap structure-specific endonuclease 1
ENSMMUG00000021517	*SCARA3*	-0.48	3.14E-02	scavenger receptor class A member 3
ENSMMUG00000001955	*C11H12orf66*	0.84	3.15E-02	KICSTOR subunit 2
ENSMMUG00000013587	*PLCD3*	-0.44	3.15E-02	phospholipase C delta 3
ENSMMUG00000022404	*APAF1*	-0.45	3.16E-02	apoptotic peptidase activating factor 1
ENSMMUG00000021189	*RF02216*	0.81	3.18E-02	NA
ENSMMUG00000042971	*CD59*	-0.45	3.18E-02	CD59 molecule (CD59 blood group)
ENSMMUG00000011305	*TMTC2*	-0.52	3.18E-02	transmembrane O-mannosyltransferase targeting cadherins 2
ENSMMUG00000011209	*BICDL1*	-0.50	3.19E-02	BICD family like cargo adaptor 1
ENSMMUG00000006984	*FBXO33*	-0.52	3.19E-02	F-box protein 33
ENSMMUG00000015929	*N/A*	-1.33	3.19E-02	N/A
ENSMMUG00000020912	*SMOX*	0.99	3.20E-02	spermine oxidase
ENSMMUG00000001690	*CNN3*	-0.46	3.22E-02	calponin 3
ENSMMUG00000021847	*MYLPF*	-0.82	3.24E-02	myosin light chain, phosphorylatable, fast skeletal muscle
ENSMMUG00000020354	*SLC3A2*	0.90	3.27E-02	solute carrier family 3 member 2
ENSMMUG00000023251	*KANK2*	-0.42	3.28E-02	KN motif and ankyrin repeat domains 2
ENSMMUG00000015125	*PBLD*	-0.43	3.30E-02	phenazine biosynthesis like protein domain containing
ENSMMUG00000010180	*SOX7*	0.64	3.30E-02	SRY-box transcription factor 7
ENSMMUG00000004103	*TXNL4B*	0.71	3.35E-02	thioredoxin like 4B
ENSMMUG00000048989	*COL11A1*	-0.49	3.35E-02	collagen type XI alpha 1 chain
ENSMMUG00000023225	*E2F6*	0.57	3.35E-02	E2F transcription factor 6
ENSMMUG00000020226	*WBS1*	-0.47	3.35E-02	WD repeat and SOCS box containing 1
ENSMMUG00000014183	*PLEKHF2*	-0.66	3.39E-02	pleckstrin homology and FYVE domain containing 2
ENSMMUG00000002242	*VEGFA*	0.74	3.42E-02	vascular endothelial growth factor A
ENSMMUG00000004577	*MECOM*	-0.65	3.42E-02	MDS1 and EVI1 complex locus
ENSMMUG00000012821	*PCYT1B*	-1.61	3.46E-02	phosphate cytidylyltransferase 1B, choline
ENSMMUG00000002404	*MINK1*	-0.42	3.47E-02	misshapen like kinase 1
ENSMMUG00000019369	*PLEKHG2*	-0.62	3.48E-02	pleckstrin homology and RhoGEF domain containing G2
ENSMMUG00000010944	*GATA6*	-0.52	3.52E-02	GATA binding protein 6
ENSMMUG00000004416	*BYSL*	0.50	3.56E-02	bystin like
ENSMMUG00000020119	*IFRD2*	0.46	3.56E-02	interferon related developmental regulator 2
ENSMMUG00000004559	*SLC16A4*	-0.60	3.59E-02	solute carrier family 16 member 4
ENSMMUG00000008710	*TFEC*	-0.86	3.59E-02	transcription factor EC
ENSMMUG00000015022	*KCND1*	-1.61	3.59E-02	potassium voltage-gated channel subfamily D member 1
ENSMMUG00000038489	*PCDH1*	0.57	3.59E-02	protocadherin 1
ENSMMUG00000031161	*GMNC*	-1.15	3.59E-02	geminin coiled-coil domain containing
ENSMMUG00000003100	*CHML*	-0.57	3.59E-02	CHM like Rab escort protein
ENSMMUG00000001895	*NDUFAF4*	0.42	3.59E-02	NADH:ubiquinone oxidoreductase complex assembly factor 4
ENSMMUG00000018843	*TMEM39A*	0.73	3.59E-02	transmembrane protein 39A
ENSMMUG00000012010	*N/A*	1.75	3.59E-02	N/A
ENSMMUG00000039762	*MYORG*	-0.62	3.62E-02	Myogenesis regulating glycosidase (putative)
ENSMMUG00000019974	*NARS*	0.50	3.62E-02	asparaginyl-tRNA synthetase 1
ENSMMUG00000013718	*TRAM2*	-0.51	3.63E-02	translocation associated membrane protein 2
ENSMMUG00000013848	*FUT8*	-0.44	3.66E-02	fucosyltransferase 8
ENSMMUG00000008101	*SPATS2L*	-0.40	3.69E-02	spermatogenesis associated serine rich 2 like

ENSMMUG00000004020	*FUT1*	1.50	3.70E-02	fucosyltransferase 1 (H blood group)
ENSMMUG00000012254	*ARHGEF12*	-0.49	3.71E-02	Rho guanine nucleotide exchange factor 12
ENSMMUG00000009511	*TRIB1*	0.95	3.75E-02	tribbles pseudokinase 1
ENSMMUG00000002116	*ASB1*	0.73	3.75E-02	ankyrin repeat and SOCS box containing 1
ENSMMUG00000004885	*BTG3*	0.43	3.75E-02	BTG anti-proliferation factor 3
ENSMMUG00000021759	*QSOX2*	0.53	3.75E-02	quiescin sulfhydryl oxidase 2
ENSMMUG00000015414	*FAM160B1*	-0.51	3.75E-02	FHF complex subunit HOOK interacting protein 2A
ENSMMUG00000020664	*FLRT2*	-0.46	3.75E-02	fibronectin leucine rich transmembrane protein 2
ENSMMUG00000005573	*LMBRD1*	-0.49	3.78E-02	LMBR1 domain containing 1
ENSMMUG00000015860	*C11H12orf29*	0.69	3.79E-02	chromosome 11 C12orf29 homolog
ENSMMUG00000018095	*EIF2S2*	0.46	3.81E-02	eukaryotic translation initiation factor 2 subunit beta
ENSMMUG00000013984	*ABLIM2*	-0.70	3.83E-02	actin binding LIM protein family member 2
ENSMMUG00000005355	*TIMP2*	-0.50	3.86E-02	TIMP metallopeptidase inhibitor 2
ENSMMUG00000001811	*FMN1*	-0.64	3.86E-02	formin 1
ENSMMUG00000001511	*SMIM3*	0.45	3.86E-02	small integral membrane protein 3
ENSMMUG00000040546	*KIF20A*	-0.89	3.86E-02	kinesin family member 20A
ENSMMUG00000020823	*VCL*	-0.53	3.86E-02	vinculin
ENSMMUG00000040229	*N/A*	-1.34	3.89E-02	N/A
ENSMMUG00000023406	*HID1*	0.56	3.89E-02	HID1 domain containing
ENSMMUG00000017232	*BMPER*	0.76	3.90E-02	BMP binding endothelial regulator
ENSMMUG00000046717	*LOC100427851*	0.92	3.92E-02	Ferritin light chain pseudogene
ENSMMUG00000016810	*PAX6*	-0.41	3.94E-02	paired box 6
ENSMMUG00000015352	*ECHDC1*	-0.51	3.96E-02	ethylmalonyl-CoA decarboxylase 1
ENSMMUG00000015925	*GRK2*	0.41	3.96E-02	G protein-coupled receptor kinase 2
ENSMMUG00000012563	*IFNAR2*	0.45	3.97E-02	interferon alpha and beta receptor subunit 2
ENSMMUG00000012243	*APBB2*	-0.40	3.97E-02	amyloid beta precursor protein binding family B member 2
ENSMMUG00000022702	*GART*	0.43	3.97E-02	phosphoribosylglycinamide formyltransferase
ENSMMUG00000003956	*FAM76A*	-0.47	3.97E-02	family with sequence similarity 76 member A
ENSMMUG00000000123	*NHSL1*	0.69	3.98E-02	NHS like 1
ENSMMUG00000021813	*NDUFAF2*	0.65	3.99E-02	NADH:ubiquinone oxidoreductase complex assembly factor 2
ENSMMUG00000047687	*RF01871*	-0.68	3.99E-02	N/A
ENSMMUG00000009926	*SERPINE1*	0.57	4.00E-02	serpin family E member 1
ENSMMUG00000009559	*MCMBP*	0.44	4.02E-02	minichromosome maintenance complex binding protein
ENSMMUG00000015540	*BTBD11*	-0.47	4.05E-02	BTB domain containing 11
ENSMMUG00000037904	*PGRMC2*	-0.46	4.05E-02	progesterone receptor membrane component 2
ENSMMUG00000000427	*BUD31*	0.51	4.07E-02	BUD31 homolog
ENSMMUG00000030046	*FNIP2*	0.82	4.07E-02	folliculin interacting protein 2
ENSMMUG00000023056	*CLCN5*	0.64	4.10E-02	chloride voltage-gated channel 5
ENSMMUG00000022060	*ARHGAP9*	1.57	4.11E-02	Rho GTPase activating protein 9
ENSMMUG00000006841	*MYOF*	-0.39	4.12E-02	myoferlin
ENSMMUG00000013782	*LAMA4*	-0.37	4.14E-02	laminin subunit alpha 4
ENSMMUG00000032084	*PARS2*	0.51	4.14E-02	prolyl-tRNA synthetase 2, mitochondrial
ENSMMUG00000021243	*ZBTB47*	-0.48	4.15E-02	zinc finger and BTB domain containing 47
ENSMMUG00000004408	*SEC11C*	0.48	4.16E-02	SEC11 homolog C, signal peptidase complex subunit
ENSMMUG00000003154	*SLC39A4*	-0.61	4.16E-02	solute carrier family 39 member 4
ENSMMUG00000000750	*RFFL*	0.53	4.16E-02	ring finger and FYVE like domain containing E3 ubiquitin protein ligase
ENSMMUG00000013888	*NOP56*	0.54	4.16E-02	NOP56 ribonucleoprotein
ENSMMUG00000002100	*MITF*	-0.59	4.17E-02	melanocyte inducing transcription factor
ENSMMUG00000011665	*MMAB*	-0.49	4.21E-02	metabolism of cobalamin associated B
ENSMMUG00000017161	*ANKRD11*	0.45	4.22E-02	ankyrin repeat domain containing 11

ENSMMUG00000040952	*TIGD7*	-1.11	4.22E-02	tigger transposable element derived 7
ENSMMUG00000009549	*CLDND1*	0.50	4.24E-02	claudin domain containing 1
ENSMMUG00000011218	*SERPINA3*	1.66	4.24E-02	serpin family A member 3
ENSMMUG00000001941	*CXXC5*	-0.42	4.25E-02	CXXC finger protein 5
ENSMMUG00000021181	*PCDH10*	-1.63	4.25E-02	protocadherin 10
ENSMMUG00000004930	*MAP3K8*	0.89	4.25E-02	mitogen-activated protein kinase kinase kinase 8
ENSMMUG00000001420	*SESN3*	-0.50	4.25E-02	sestrin 3
ENSMMUG00000023149	*MFAP2*	-0.79	4.25E-02	microfibril associated protein 2
ENSMMUG00000009394	*LRRC25*	1.42	4.25E-02	leucine rich repeat containing 25
ENSMMUG00000005469	*ERMP1*	-0.55	4.29E-02	endoplasmic reticulum metallopeptidase 1
ENSMMUG00000046631	*RFLNA*	-0.59	4.29E-02	refilin A
ENSMMUG00000005959	*GK*	0.74	4.30E-02	glycerol kinase
ENSMMUG00000020147	*CSRNP1*	0.76	4.30E-02	glycerol kinase
ENSMMUG00000013716	*EFHC1*	-0.43	4.34E-02	cysteine and serine rich nuclear protein 1
ENSMMUG00000028670	*FGF2*	0.57	4.34E-02	EF-hand domain containing 1
ENSMMUG00000007419	*GASK1B*	-0.70	4.37E-02	fibroblast growth factor 2
ENSMMUG00000008547	*CYTB*	-0.51	4.37E-02	golgi associated kinase 1B
ENSMMUG00000019238	*RNPS1*	0.42	4.37E-02	Cytochrome b
ENSMMUG00000019634	*RRP12*	0.67	4.40E-02	RNA binding protein with serine rich domain 1
ENSMMUG00000009833	*SHC4*	1.14	4.40E-02	ribosomal RNA processing 12 homolog
ENSMMUG00000019993	*SESN2*	1.00	4.42E-02	SHC adaptor protein 4
ENSMMUG00000047956	*CDC42SE2*	0.47	4.43E-02	sestrin 2
ENSMMUG00000001134	*LOC709988*	-0.43	4.44E-02	CDC42 small effector 2
ENSMMUG00000018870	*C15H9orf64*	0.55	4.47E-02	cyclin-dependent kinase inhibitor 2A
ENSMMUG00000015758	*GLIS2*	-0.80	4.51E-02	chromosome 15 C9orf64 homolog
ENSMMUG00000015774	*NPM3*	0.45	4.51E-02	GLIS family zinc finger 2
ENSMMUG00000020359	*SPTBN2*	-0.62	4.51E-02	nucleophosmin/nucleoplasmin 3
ENSMMUG00000017133	*SLC12A6*	-0.43	4.51E-02	spectrin beta, non-erythrocytic 2
ENSMMUG00000020935	*N/A*	0.53	4.52E-02	solute carrier family 12 member 6
ENSMMUG00000019269	*ACTR8*	0.44	4.52E-02	N/A
ENSMMUG00000011513	*VCAN*	-0.55	4.57E-02	actin related protein 8
ENSMMUG00000014122	*CAV1*	-0.53	4.62E-02	versican
ENSMMUG00000000873	*TRPV2*	-0.50	4.64E-02	caveolin 1
ENSMMUG00000015614	*RABGGTB*	0.50	4.66E-02	transient receptor potential cation channel subfamily V member 2
ENSMMUG00000007367	*RARB*	-0.79	4.66E-02	Rab geranylgeranyltransferase subunit beta
ENSMMUG00000030573	*ADIRF*	-0.43	4.67E-02	retinoic acid receptor beta
ENSMMUG00000020074	*PKDCC*	-0.47	4.69E-02	adipogenesis regulatory factor
ENSMMUG00000019746	*YARS*	0.48	4.69E-02	protein kinase domain containing, cytoplasmic
ENSMMUG00000013359	*SLC11A1*	-0.60	4.74E-02	tyrosyl-tRNA synthetase 1
ENSMMUG00000006553	*DVL3*	-0.45	4.75E-02	solute carrier family 11 member 1
ENSMMUG00000020965	*EFNB1*	-0.42	4.77E-02	dishevelled segment polarity protein 3
ENSMMUG00000006548	*SH2D4A*	-0.60	4.79E-02	ephrin B1
ENSMMUG00000000129	*KIAA1549*	-0.53	4.81E-02	SH2 domain containing 4A
ENSMMUG00000021053	*ANXA6*	-0.43	4.81E-02	KIAA1549
ENSMMUG00000041923	*NFKBIE*	0.54	4.82E-02	annexin A6
ENSMMUG00000022689	*NANS*	0.45	4.83E-02	NFKB inhibitor epsilon
ENSMMUG00000015072	*PREX1*	0.56	4.83E-02	N-acetylneuraminate synthase
ENSMMUG00000041404	*LYNX1*	-0.40	4.84E-02	phosphatidylinositol-3,4,5-trisphosphate dependent Rac exchange factor 1
ENSMMUG00000042262	*PMP22*	-0.42	4.85E-02	Ly6/neurotoxin 1
ENSMMUG00000002206	*NOS1AP*	-1.11	4.85E-02	peripheral myelin protein 22
ENSMMUG00000003036	*GMIP*	-0.72	4.85E-02	nitric oxide synthase 1 adaptor protein
ENSMMUG00000019393	*TNS3*	-0.39	4.86E-02	GEM interacting protein

ENSMMUG00000021553	*DHRS13*	0.90	4.90E-02	tensin 3
ENSMMUG00000044197	*INHBA*	-0.73	4.91E-02	dehydrogenase/reductase 13
ENSMMUG00000006069	*FNDC11*	-0.81	4.95E-02	inhibin subunit beta A
ENSMMUG00000019983	*IL6*	1.36	4.98E-02	fibronectin type III domain containing 11
ENSMMUG00000001226	*HMGCS1*	-3.10	4.98E-02	interleukin 6

Table S2. Complete list of the reversion effect exert by 10 µM treatment over 15 µM 7KCh-altered genes in mRPE cells. Log2FC and FDR values correspond to the comparison of the combined treatment with 7KCh alone.

Ensemble ID	Gene ID	Log2FC	FDR	Gene description
ENSMMUG00000001618	*ATP6V0D2*	-4.77	4.67E-60	ATPase H+ transporting V0 subunit D2
ENSMMUG00000012185	*ATF3*	-2.36	2.29E-30	activating transcription factor 3
ENSMMUG00000000208	*NRG1*	-2.28	4.08E-30	neuregulin 1
ENSMMUG00000011593	*RGCC*	-3.57	1.62E-29	regulator of cell cycle
ENSMMUG00000000006	*NEURL3*	-4.23	8.12E-28	neuralized E3 ubiquitin protein ligase 3
ENSMMUG00000019777	*IL1A*	-3.07	7.81E-25	interleukin 1 alpha
ENSMMUG00000010189	*TMEM71*	-2.68	5.16E-23	transmembrane protein 71
ENSMMUG00000001511	*KIF20A*	2.00	1.62E-21	kinesin family member 20A
ENSMMUG00000009852	*CASP1*	-2.10	4.20E-21	caspase-1
ENSMMUG00000014176	*CYTIP*	-2.69	7.14E-21	cytohesin 1 interacting protein
ENSMMUG00000023801	*LCN2*	-2.65	2.27E-19	lipocalin 2
ENSMMUG00000045506	*MAFF*	-1.79	3.47E-19	MAF bZIP transcription factor F
ENSMMUG00000014430	*FOSB*	-4.42	5.08E-19	FosB proto-oncogene, AP-1 transcription factor subunit
ENSMMUG00000041958	*BHLHA15*	-4.03	2.53E-18	basic helix-loop-helix family member a15
ENSMMUG00000019326	*CTH*	-1.71	2.83E-18	cystathionine gamma-lyase
ENSMMUG00000007037	*UPP1*	-1.71	3.54E-18	uridine phosphorylase 1
ENSMMUG00000044653	*CLDN4*	-2.58	6.60E-17	claudin 4
ENSMMUG00000008335	*SAT1*	-1.60	1.42E-16	spermidine/spermine N1-acetyltransferase 1
ENSMMUG00000003708	*PDE7B*	-2.11	1.45E-16	phosphodiesterase 7B
ENSMMUG00000013290	*LURAP1L*	-1.92	9.56E-16	leucine rich adaptor protein 1 like
ENSMMUG00000048596	*CXCL2*	-2.07	1.94E-14	C-X-C motif chemokine ligand 2
ENSMMUG00000009223	*PPP1R15A*	-1.53	2.09E-14	protein phosphatase 1 regulatory subunit 15A
ENSMMUG00000006867	*MYPN*	-2.62	2.88E-14	myopalladin
ENSMMUG00000006088	*KLF4*	-2.12	3.03E-14	Kruppel like factor 4
ENSMMUG00000004504	*KITLG*	-2.23	4.19E-14	KIT ligand
ENSMMUG00000004403	*ID1*	1.46	6.12E-14	inhibitor of DNA binding 1, HLH protein
ENSMMUG00000020806	*ABCC9*	-2.05	7.20E-14	ATP binding cassette subfamily C member 9
ENSMMUG00000019157	*ASNS*	-1.42	8.22E-14	asparagine synthetase (glutamine-hydrolyzing)
ENSMMUG00000008869	*TNFAIP3*	-1.77	2.26E-13	TNF alpha induced protein 3
ENSMMUG00000020157	*GBP6*	-1.94	3.77E-12	guanylate binding protein family member 6
ENSMMUG00000015329	*MX1*	-2.29	4.73E-12	MX dynamin like GTPase 1
ENSMMUG00000003721	*KLF15*	-2.49	1.07E-11	Kruppel like factor 15
ENSMMUG00000011868	*PSAT1*	-1.28	1.93E-11	phosphoserine aminotransferase 1
ENSMMUG00000010927	*TNFAIP6*	-2.04	2.54E-11	TNF alpha induced protein 6
ENSMMUG00000016040	*RND3*	-1.27	2.64E-11	Rho family GTPase 3
ENSMMUG00000023089	*PHGDH*	-1.46	2.64E-11	phosphoglycerate dehydrogenase
ENSMMUG00000012600	*TRIB3*	-1.23	1.93E-10	tribbles pseudokinase 3
ENSMMUG00000019983	*IL6*	-2.28	1.16E-09	interleukin 6
ENSMMUG00000005947	*SAMD11*	1.33	2.24E-09	sterile alpha motif domain containing 11
ENSMMUG00000016938	*DFNA5*	-1.21	2.60E-09	gasdermin E
ENSMMUG00000019996	*ENC1*	1.21	2.90E-09	ectodermal-neural cortex 1
ENSMMUG00000023257	*NFIL3*	-1.41	4.91E-09	nuclear factor, interleukin 3 regulated
ENSMMUG00000042454	*PHLDA1*	-1.15	4.91E-09	pleckstrin homology like domain family A member 1
ENSMMUG00000008033	*IFRD1*	-1.16	6.55E-09	interferon related developmental regulator 1
ENSMMUG00000000947	*MSC*	-1.16	6.56E-09	musculin
ENSMMUG00000022481	*CYP1A1*	-2.54	7.50E-09	cytochrome P450 family 1 subfamily A member 1
ENSMMUG00000015158	*SLC6A9*	-1.32	8.81E-09	solute carrier family 6 member 9
ENSMMUG00000010601	*PRG2*	-1.58	8.81E-09	proteoglycan 2, pro eosinophil major basic protein

ENSMMUG00000032156	*C3AR1*	-1.53	1.20E-08	complement C3a receptor 1
ENSMMUG00000009289	*CBS*	-1.47	1.20E-08	cystathionine beta-synthase
ENSMMUG00000001819	*ISG15*	-1.32	1.29E-08	ISG15 ubiquitin like modifier
ENSMMUG00000000567	*BAMBI*	1.20	2.30E-08	BMP and activin membrane bound inhibitor
ENSMMUG00000006269	*CSTA*	-2.05	3.69E-08	cystatin A
ENSMMUG00000009084	*TM6SF1*	-2.72	3.98E-08	transmembrane 6 superfamily member 1
ENSMMUG00000019993	*SESN2*	-1.05	1.35E-07	sestrin 2
ENSMMUG00000045219	*CEBPB*	-1.20	1.58E-07	CCAAT enhancer binding protein beta
ENSMMUG00000017169	*FAM107B*	-1.33	1.58E-07	family with sequence similarity 107 member B
ENSMMUG00000011845	*HK2*	-1.09	2.31E-07	hexokinase 2
ENSMMUG00000016848	*SLCO4A1*	-1.06	2.38E-07	solute carrier organic anion transporter family member 4A1
ENSMMUG00000013610	*ETV5*	-1.04	2.50E-07	ETS variant transcription factor 5
ENSMMUG00000022873	*SERPINB8*	-1.19	2.75E-07	serpin family B member 8
ENSMMUG00000007090	*DOK5*	-1.10	2.90E-07	docking protein 5
ENSMMUG00000019992	*GRB10*	-1.63	3.32E-07	growth factor receptor bound protein 10
ENSMMUG00000008140	*SGK1*	-1.01	4.12E-07	serum/glucocorticoid regulated kinase 1
ENSMMUG00000000265	*ADAMTS9*	-1.34	5.22E-07	ADAM metallopeptidase with thrombospondin type 1 motif 9
ENSMMUG00000039576	*N/A*	-1.00	6.78E-07	N/A
ENSMMUG00000013797	*GFPT1*	-1.01	8.26E-07	glutamine--fructose-6-phosphate transaminase 1
ENSMMUG00000019849	*BMP2*	-1.30	9.44E-07	bone morphogenetic protein 2
ENSMMUG00000002052	*SPSB1*	-1.22	9.48E-07	splA/ryanodine receptor domain and SOCS box containing 1
ENSMMUG00000004577	*VEGFA*	-0.97	1.15E-06	vascular endothelial growth factor A
ENSMMUG00000009914	*SMAD9*	1.30	1.82E-06	SMAD family member 9
ENSMMUG00000013059	*GPR180*	-0.97	1.83E-06	G protein-coupled receptor 180
ENSMMUG00000020608	*ABCA1*	-0.96	3.65E-06	ATP binding cassette subfamily A member 1
ENSMMUG00000040573	*PMAIP1*	-1.13	5.38E-06	phorbol-12-myristate-13-acetate-induced protein 1
ENSMMUG00000001952	*SRGAP1*	-1.31	5.76E-06	SLIT-ROBO Rho GTPase activating protein 1
ENSMMUG00000001031	*ABHD5*	-0.91	7.26E-06	abhydrolase domain containing 5, lysophosphatidic acid acyltransferase
ENSMMUG00000002517	*PTPDC1*	-2.16	9.59E-06	protein tyrosine phosphatase domain containing 1
ENSMMUG00000021590	*PCK2*	-0.96	1.12E-05	phosphoenolpyruvate carboxykinase 2, mitochondrial
ENSMMUG00000021759	*TRIB1*	-1.01	1.20E-05	tribbles pseudokinase 1
ENSMMUG00000043586	*ID3*	0.93	1.21E-05	inhibitor of DNA binding 3, HLH protein
ENSMMUG00000007419	*FGF2*	-1.01	1.21E-05	fibroblast growth factor 2
ENSMMUG00000041399	*UBAP1L*	-2.03	1.26E-05	ubiquitin associated protein 1 like
ENSMMUG00000002933	*NR1D1*	-1.30	1.33E-05	nuclear receptor subfamily 1 group D member 1
ENSMMUG00000010514	*SLC6A20*	0.92	1.37E-05	solute carrier family 6 member 20
ENSMMUG00000014219	*OSGIN1*	-1.04	1.47E-05	oxidative stress induced growth inhibitor 1
ENSMMUG00000005217	*HSPA5*	-0.86	1.69E-05	heat shock protein family A (Hsp70) member 5
ENSMMUG00000012895	*PYCR1*	-0.92	2.08E-05	pyrroline-5-carboxylate reductase 1
ENSMMUG00000011999	*SLC38A1*	-0.87	2.97E-05	solute carrier family 38 member 1
ENSMMUG00000048076	*TNFRSF10B*	-0.88	2.97E-05	TNF receptor superfamily member 10b
ENSMMUG00000022163	*SLC1A5*	-0.86	3.18E-05	solute carrier family 1 member 5
ENSMMUG00000018907	*ERFE*	-1.12	3.39E-05	erythroferrone
ENSMMUG00000017232	*BMPER*	-1.37	3.56E-05	BMP binding endothelial regulator
ENSMMUG00000045851	*N/A*	-0.86	4.54E-05	N/A
ENSMMUG00000007659	*XPOT*	-0.87	4.68E-05	exportin for tRNA
ENSMMUG00000030046	*FNIP2*	-0.86	5.00E-05	folliculin interacting protein 2
ENSMMUG00000005742	*CDCP1*	-0.99	5.23E-05	CUB domain containing protein 1
ENSMMUG00000009063	*ETS2*	-0.84	5.27E-05	ETS proto-oncogene 2, transcription factor

ENSMMUG00000018192	*RORA*	-1.24	5.41E-05	RAR related orphan receptor A
ENSMMUG00000009238	*IL1RL1*	-1.43	5.80E-05	interleukin 1 receptor like 1
ENSMMUG00000041498	*TRAF1*	-0.92	5.92E-05	TNF receptor associated factor 1
ENSMMUG00000016223	*MYCL*	-1.11	8.82E-05	MYCL proto-oncogene, bHLH transcription factor
ENSMMUG00000023265	*AKAP12*	-0.82	9.82E-05	A-kinase anchoring protein 12
ENSMMUG00000031911	*KRT18*	-0.83	1.03E-04	keratin 18
ENSMMUG00000014542	*CHAC1*	-1.06	1.17E-04	ChaC glutathione specific gamma-glutamylcy-clotransferase 1
ENSMMUG00000006127	*CEBPG*	-0.87	1.23E-04	CCAAT enhancer binding protein gamma
ENSMMUG00000019072	*DAAM2*	0.81	1.55E-04	dishevelled associated activator of morphogenesis 2
ENSMMUG00000044497	*C15orf48*	-1.21	1.55E-04	chromosome 15 open reading frame 48
ENSMMUG00000019850	*ALOXE3*	-1.78	1.56E-04	arachidonate lipoxygenase 3
ENSMMUG00000021837	*SARS1*	-0.82	1.69E-04	seryl-tRNA synthetase 1
ENSMMUG00000040972	*KCNK3*	1.24	2.31E-04	potassium two pore domain channel subfamily K member 3
ENSMMUG00000017327	*SLAMF7*	-2.39	2.59E-04	SLAM family member 7
ENSMMUG00000012010	*TMEM39A*	-0.79	2.61E-04	transmembrane protein 39A
ENSMMUG00000010594	*PTGFR*	-0.99	2.96E-04	prostaglandin F receptor
ENSMMUG00000006971	*ARF3*	-0.93	3.22E-04	ADP ribosylation factor 3
ENSMMUG00000014952	*NUP210L*	-0.98	3.51E-04	Nucleoporin 210 like
ENSMMUG00000019126	*PNP*	-0.76	3.65E-04	purine nucleoside phosphorylase
ENSMMUG00000013407	*ADAMTS1*	-0.95	4.34E-04	ADAM metallopeptidase with thrombospondin type 1 motif 1
ENSMMUG00000021189	*RF02216*	-1.06	4.70E-04	N/A
ENSMMUG00000015817	*TMTC1*	0.77	5.14E-04	transmembrane O-mannosyltransferase targeting cadherins 1
ENSMMUG00000016418	*CBX4*	-0.79	5.80E-04	chromobox 4
ENSMMUG00000009739	*MTHFD2*	-0.77	6.06E-04	methylenetetrahydrofolate dehydrogenase (NADP+ dependent) 2, methenyltetrahydrofolate cyclohydro-lase
ENSMMUG00000016074	*LFNG*	1.19	7.22E-04	LFNG O-fucosylpeptide 3-beta-N-acetylglucosami-nyltransferase
ENSMMUG00000011294	*LSR*	-0.72	8.12E-04	lipolysis stimulated lipoprotein receptor
ENSMMUG00000010531	*PTHLH*	-1.81	8.80E-04	parathyroid hormone like hormone
ENSMMUG00000004719	*GARS1*	-0.73	8.95E-04	glycyl-tRNA synthetase 1
ENSMMUG00000000664	*RRAS2*	-0.74	9.23E-04	RAS related 2
ENSMMUG00000007798	*CLMP*	-1.30	1.04E-03	CXADR like membrane protein
ENSMMUG00000012223	*BCAT1*	-1.00	1.04E-03	branched chain amino acid transaminase 1
ENSMMUG00000021785	*SHMT2*	-0.70	1.08E-03	serine hydroxymethyltransferase 2
ENSMMUG00000018053	*ZBTB21*	-0.71	1.08E-03	zinc finger and BTB domain containing 21
ENSMMUG00000013640	*ZBTB43*	-0.72	1.13E-03	zinc finger and BTB domain containing 43
ENSMMUG00000014498	*CACNA1E*	-1.24	1.26E-03	calcium voltage-gated channel subunit alpha1 E
ENSMMUG00000019974	*NARS1*	-0.71	1.29E-03	asparaginyl-tRNA synthetase 1
ENSMMUG00000005875	*HSPA9*	-0.66	1.33E-03	heat shock protein family A (Hsp70) member 9
ENSMMUG00000003292	*SLC38A10*	0.71	1.33E-03	solute carrier family 38 member 10
ENSMMUG00000019018	*SMAD6*	0.94	1.36E-03	SMAD family member 6
ENSMMUG00000017631	*NA*	-0.77	1.45E-03	secreted protein
ENSMMUG00000022066	*UTP25*	-0.78	1.45E-03	UTP25 small subunit processor component
ENSMMUG00000007906	*CDC42EP3*	-0.72	1.51E-03	CDC42 effector protein 3
ENSMMUG00000018058	*ABCG1*	-0.73	1.54E-03	ATP binding cassette subfamily G member 1
ENSMMUG00000013865	*SLC25A47*	-0.71	1.54E-03	solute carrier family 25 member 47
ENSMMUG00000012634	*GTF2F2*	-0.69	1.57E-03	general transcription factor IIF subunit 2
ENSMMUG00000016294	*SYCP2*	-1.02	1.87E-03	synaptonemal complex protein 2
ENSMMUG00000019270	*MTO1*	-0.69	1.93E-03	mitochondrial tRNA translation optimization 1

ENSMMUG00000010147	*PLA2G15*	0.82	2.04E-03	phospholipase A2 group XV
ENSMMUG00000042787	*N/A*	-2.55	2.04E-03	N/A
ENSMMUG00000040286	*N/A*	1.10	2.16E-03	N/A
ENSMMUG00000018116	*JAG1*	-0.89	2.17E-03	jagged canonical Notch ligand 1
ENSMMUG00000005778	*FOXC1*	-1.03	2.63E-03	forkhead box C1
ENSMMUG00000041773	*FABP5*	-0.66	2.80E-03	fatty acid binding protein 5
ENSMMUG00000008094	*DUSP4*	-1.00	3.49E-03	dual specificity phosphatase 4
ENSMMUG00000008890	*GTPBP2*	-0.67	3.67E-03	GTP binding protein 2
ENSMMUG00000000511	*CARS1*	-0.64	4.11E-03	cysteinyl-tRNA synthetase 1
ENSMMUG00000019514	*UAP1*	-0.67	4.24E-03	UDP-N-acetylglucosamine pyrophosphorylase 1
ENSMMUG00000000123	*NHSL1*	-0.86	4.27E-03	NHS like 1
ENSMMUG00000021074	*RASGEF1B*	-0.88	4.27E-03	RasGEF domain family member 1B
ENSMMUG00000004628	*IARS1*	-0.63	4.28E-03	isoleucyl-tRNA synthetase 1
ENSMMUG00000003587	*KLRC1*	-1.33	5.12E-03	killer cell lectin like receptor C1
ENSMMUG00000008539	*UROS*	0.63	5.47E-03	uroporphyrinogen III synthase
ENSMMUG00000014175	*SPRY4*	-0.90	5.78E-03	sprouty RTK signaling antagonist 4
ENSMMUG00000003585	*ALG5*	-0.66	6.95E-03	ALG5 dolichyl-phosphate beta-glucosyltransferase
ENSMMUG00000015185	*ERN1*	-0.66	7.18E-03	endoplasmic reticulum to nucleus signaling 1
ENSMMUG00000008653	*FOSL1*	-0.69	7.18E-03	FOS like 1, AP-1 transcription factor subunit
ENSMMUG00000006548	*KIAA1549*	0.80	7.94E-03	KIAA1549
ENSMMUG00000001048	*EAF2*	-0.81	8.77E-03	ELL associated factor 2
ENSMMUG00000011209	*BICDL1*	0.70	9.15E-03	BICD family like cargo adaptor 1
ENSMMUG00000007400	*SIAH1*	-0.63	9.25E-03	siah E3 ubiquitin protein ligase 1
ENSMMUG00000001005	*DDX21*	-0.61	9.78E-03	DExD-box helicase 21
ENSMMUG00000013531	*IPO5*	-0.58	1.09E-02	importin 5
ENSMMUG00000008003	*LRRC8C*	-0.59	1.17E-02	leucine rich repeat containing 8 VRAC subunit C
ENSMMUG00000020365	*ARHGAP18*	-0.61	1.18E-02	Rho GTPase activating protein 18
ENSMMUG00000009833	*SHC4*	-1.12	1.22E-02	SHC adaptor protein 4
ENSMMUG00000020147	*CSRNP1*	-0.68	1.26E-02	cysteine and serine rich nuclear protein 1
ENSMMUG00000002116	*BTG3*	-0.59	1.29E-02	BTG anti-proliferation factor 3
ENSMMUG00000004020	*FUT1*	-1.23	1.30E-02	fucosyltransferase 1 (H blood group)
ENSMMUG00000010813	*GTPBP4*	-0.58	1.36E-02	GTP binding protein 4
ENSMMUG00000015774	*NPM3*	-0.58	1.47E-02	nucleophosmin/nucleoplasmin 3
ENSMMUG00000024570	*LOC114677864*	-1.22	1.58E-02	small nucleolar RNA SNORA70
ENSMMUG00000039960	*N/A*	-1.62	1.70E-02	N/A
ENSMMUG00000022394	*EGFR*	-0.56	1.98E-02	epidermal growth factor receptor
ENSMMUG00000018773	*TM7SF2*	0.58	2.08E-02	transmembrane 7 superfamily member 2
ENSMMUG00000003273	*BCAM*	0.62	2.27E-02	basal cell adhesion molecule (Lutheran blood group)
ENSMMUG00000029011	*UBXN8*	-0.70	2.29E-02	UBX domain protein 8
ENSMMUG00000020839	*SEPHS2*	-0.65	2.33E-02	selenophosphate synthetase 2
ENSMMUG00000003288	*GPT2*	-0.64	2.35E-02	glutamic--pyruvic transaminase 2
ENSMMUG00000020408	*HPGD*	0.80	2.48E-02	15-hydroxyprostaglandin dehydrogenase
ENSMMUG00000004706	*IGSF9*	0.57	2.49E-02	immunoglobulin superfamily member 9
ENSMMUG00000009012	*FAM3A*	0.56	2.50E-02	FAM3 metabolism regulating signaling molecule A
ENSMMUG00000022060	*ARHGAP9*	-1.86	2.55E-02	Rho GTPase activating protein 9
ENSMMUG00000014601	*MYC*	-0.56	2.55E-02	MYC proto-oncogene, bHLH transcription factor
ENSMMUG00000019774	*MTHFD1L*	-0.54	2.72E-02	methylenetetrahydrofolate dehydrogenase (NADP+ dependent) 1 like
ENSMMUG00000023225	*E2F6*	-0.69	2.83E-02	E2F transcription factor 6
ENSMMUG00000013848	*FUT8*	0.57	3.03E-02	fucosyltransferase 8
ENSMMUG00000013984	*ABLIM2*	0.81	3.15E-02	actin binding LIM protein family member 2
ENSMMUG00000009112	*TLR4*	-0.80	3.17E-02	toll like receptor 4
ENSMMUG00000015917	*ST6GALNAC4*	-0.56	3.23E-02	ST6 N-acetylgalactosaminide alpha-2,6-sialyltransferase 4

ENSMMUG00000030891	*HYOU1*	-0.53	3.42E-02	hypoxia up-regulated 1
ENSMMUG00000016290	*WWC3*	0.52	3.46E-02	WWC family member 3
ENSMMUG00000010685	*SPCS2*	-0.53	3.47E-02	signal peptidase complex subunit 2
ENSMMUG00000017946	*ROGDI*	0.52	3.68E-02	rogdi atypical leucine zipper
ENSMMUG00000015968	*CMPK2*	-0.89	3.70E-02	cytidine/uridine monophosphate kinase 2
ENSMMUG00000023390	*TAX1BP3*	0.53	3.77E-02	N/A
ENSMMUG00000008100	*IRF1*	-0.53	3.87E-02	interferon regulatory factor 1
ENSMMUG00000038480	*EIF4EBP1*	-0.51	4.12E-02	N/A
ENSMMUG00000042262	*NOS1AP*	0.96	4.17E-02	nitric oxide synthase 1 adaptor protein
ENSMMUG00000005670	*RPS6KA2*	-0.65	4.20E-02	ribosomal protein S6 kinase A2
ENSMMUG00000012511	*GNL3*	-0.53	4.26E-02	G protein nucleolar 3
ENSMMUG00000011328	*CPEB1*	-0.62	4.40E-02	cytoplasmic polyadenylation element binding protein 1
ENSMMUG00000001283	*TP53I3*	-0.51	4.43E-02	tumor protein p53 inducible protein 3
ENSMMUG00000001172	*SOHLH2*	-1.29	4.49E-02	spermatogenesis and oogenesis specific basic helix-loop-helix 2
ENSMMUG00000020354	*SLC3A2*	-0.48	4.52E-02	solute carrier family 3 member 2
ENSMMUG00000021653	*AARS1*	-0.51	4.78E-02	alanyl-tRNA synthetase 1
ENSMMUG00000001618	*ATP6V0D2*	-4.77	4.67E-60	ATPase H+ transporting V0 subunit D2
